# African-centric TP53 variant increases iron accumulation and bacterial pathogenesis but improves response to malaria toxin

**DOI:** 10.1038/s41467-019-14151-9

**Published:** 2020-01-24

**Authors:** Kumar Sachin Singh, Julia I-Ju Leu, Thibaut Barnoud, Prashanthi Vonteddu, Keerthana Gnanapradeepan, Cindy Lin, Qin Liu, James C. Barton, Andrew V. Kossenkov, Donna L. George, Maureen E. Murphy, Farokh Dotiwala

**Affiliations:** 1 Vaccine and Immunotherapy Center, The Wistar Institute, Philadelphia, PA 19104 USA; 2 Department of Genetics, The Raymond and Ruth Perelman School of Medicine at the University of Pennsylvania, Philadelphia, PA 19104 USA; 3 Program in Molecular and Cellular Oncogenesis, The Wistar Institute, Philadelphia, PA 19104 USA; 4 Graduate Group in Biochemistry and Molecular Biophysics, The Raymond and Ruth Perelman School of Medicine at the University of Pennsylvania, Philadelphia, PA 19104 USA; 5 Program in Immunology, Microenvironment and Metastasis, The Wistar Institute, Philadelphia, PA 19104 USA; 6 Southern Iron Disorders Center, Birmingham AL 35209 USA and Department of Medicine, University of Alabama at Birmingham, Birmingham, AL 35294 USA; 7 Bioinformatics Facility, The Wistar Institute, Philadelphia, PA 19104 USA

**Keywords:** Monocytes and macrophages, Bacterial host response, Parasite host response

## Abstract

A variant at amino acid 47 in human TP53 exists predominantly in individuals of African descent. P47S human and mouse cells show increased cancer risk due to defective ferroptosis. Here, we show that this ferroptotic defect causes iron accumulation in P47S macrophages. This high iron content alters macrophage cytokine profiles, leads to higher arginase level and activity, and decreased nitric oxide synthase activity. This leads to more productive intracellular bacterial infections but is protective against malarial toxin hemozoin. Proteomics of macrophages reveal decreased liver X receptor (LXR) activation, inflammation and antibacterial defense in P47S macrophages. Both iron chelators and LXR agonists improve the response of P47S mice to bacterial infection. African Americans with elevated saturated transferrin and serum ferritin show higher prevalence of the P47S variant (OR = 1.68 (95%CI 1.07–2.65) p = 0.023), suggestive of its role in iron accumulation in humans. This altered macrophage phenotype may confer an advantage in malaria-endemic sub-Saharan Africa.

## Abstract (web summary)

A polymorphism in human TP53 (P47S) that predominantly exists in individuals of African descent affects ferroptosis. Here, the authors show that this results in iron accumulation in macrophages leading to more productive infection by intracellular bacteria but improved anti-inflammatory response to the malarial toxin hemozoin.

## Introduction

Macrophages are important in maintaining homeostasis in tissues, where they can sense and respond to infection and tissue injury^1–3^. During a microbial infection, macrophages detect pathogen-associated molecular patterns (PAMPs) using their pattern recognition receptors (PRRs). The macrophage adaptive response follows attempts to destroy or contain the pathogens and limits tissue injury by excessive immune response^4^. In addition to infection, macrophages can sense and respond to other perturbations such as cancers, chemicals, stress, or inflammation by adopting a pro- or anti-inflammatory role^2,3,5^. The control of iron homeostasis is one of the major functions of macrophages in a healthy human^6^ and in response to microbial infections^7–9^.

In mammals, hemoglobin within red blood cells (RBCs) is the largest pool of iron (4–5 g)^10^. Release of this iron into circulation by RBC senescence or damage can cause (a) iron cytotoxicity and programmed cell death by ferroptosis^11^ and (b) catalysis of Fenton reaction by free iron that converts reactive oxygen species to hydroxyl radicals that damage lipids, DNA, and proteins^12,13^. Hence, in healthy individuals erythrophagocytic macrophages prevent hemoglobin release from RBCs and recycle the iron to the erythroid progenitors^6^. Iron is essential for formation of bacterial iron–sulfur cluster enzymes and proliferation. Some hemolytic pathogens have evolved mechanisms of liberating iron from hemoglobin, whereas most bacteria such as *Listeria monocytogenes* (Lm) and *Mycobacterium tuberculosis* (MTB) produce or transport ferric siderophores, which are small proteins capable of chelating iron from transferrin^14–16^. Overall, in addition to mitigating iron toxicity, macrophages regulate iron distribution as an innate immune response to invading microbes^17,18^.

*TP53* is a well-studied tumor suppressor gene that contains several functionally significant coding region polymorphisms that alter its function^19^. The P47S (hereafter S47) polymorphism in *TP53* has an allele frequency of ~ 1.2% in African Americans and is associated with decreased ability to regulate specific *TP53* target genes associated with ferroptosis; human and mouse cells containing the S47 variant are defective for ferroptotic cell death^20^. Here, we investigate the consequences of this ferroptotic defect on iron accumulation, macrophage function, and on the progression of bacterial infection. Our data indicate that the ferroptotic defect in S47 mice leads to increased iron accumulation in macrophages, leading them to be skewed toward an anti-inflammatory phenotype (M2). This renders mice with poorer response to bacterial infections like Lm, but with improved response to the malarial toxin hemozoin. These findings suggest that the ferroptotic defect in S47 humans and mice may influence the severity of certain infectious diseases.

## Results

### Iron accumulation in S47 mouse macrophages

**Fig. 1:**
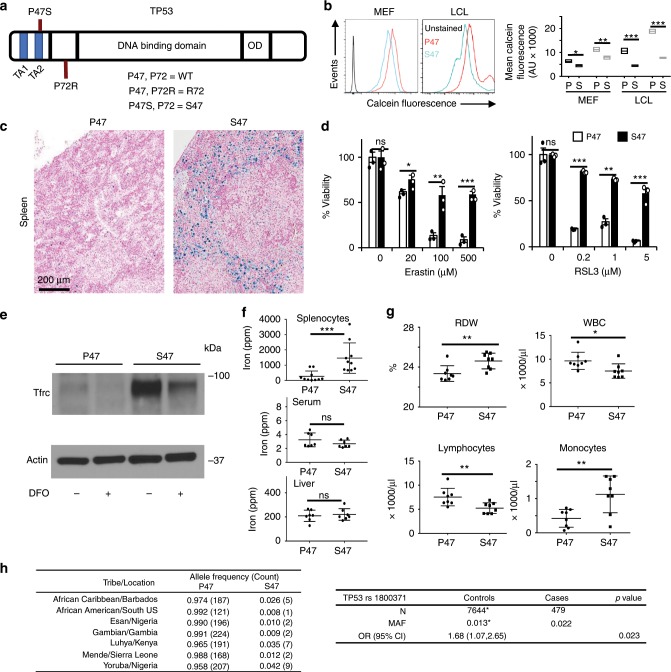
Iron accumulation in mice containing the S47 variant. **a** Domain model of *TP53* showing the positions of S47 P47 and R72 SNPs studied in this paper. **b** Iron levels in P47 and S47 human lymphoblastoid cell lines and mouse embryonic fibroblasts measured by loss of calcein fluorescence. Mean fluorescence intensity reflects iron levels in untreated (black) or 100 μm DFO-treated (gray) MEFs and LCLs (*n* = 3 biological replicates). Error bars represent means ± s.e.m. **P < 0.01, **P < 0.01, *P < 0.05, by unpaired Student’s *t* test, relative to P47 mice. **c** Formalin-fixed sections of male S47 and P47 mouse spleens stained by Prussian Blue; representative images are of six biological replicates per group with 5–7 fields per sample. **d** Percent viability of P47 (white) and S47 (black) monocyte-derived macrophages, 48 h after treatment with ferroptosis inducers Erastin and RSL3. (*n* = 3 biological replicates each with three technical replicates). Error bars represent means ± s.e.m. ***P < 0.001, **P < 0.01, *P < 0.05, ns—not significant; by unpaired Student’s *t* test. **e** Relative levels of the iron transport protein Tfrc in P47 and S47 mouse MDMs measured by immunoblot with or without DFO treatment (representative blot of three technical replicates). **f** ICP-MS shows significantly higher iron content in S47 male mouse splenocytes (*n* = 10 biological replicates). Error bars represent means ± s.e.m. ***P < 0.001, **P < 0.01, *P < 0.05, ns—not significant; by unpaired Student’s *t* test, relative to P47 mice. **g** CBC profiles in S47 and P47 male mice (*n* = 8 biological replicates). Error bars represent means ± s.e.m. **P < 0.01, *P < 0.05, by unpaired Student’s *t* test, relative to P47 mice. **h** Allelic frequencies for the S47 SNP in populations of African descent; data are obtained from the 1000 Genomes Project (left panel). Associations between *TP53* rs1800371 (S47) in individuals with high serum ferritin level and/or high saturated transferrin receptor in African Americans from the HEIRS database (right panel). (MAF = minor allele frequency, OR (95% CI) = odds ratio (95% confidence interval), * from^25^. Source data are provided as a Source Data file.

Unlike most cancer-associated genes, *TP53* is distinguished by multiple coding region polymorphisms, several of which can have a marked impact on p53 function. The P47S variant (hereafter called S47) is restricted to individuals of African descent, and is located in the second transactivation domain of the p53 protein (Fig. 1a). This variant is located in exon 4, and is linked to the P72 variant at codon 72 of p53 (P72, Fig. 1a)^21^. We previously generated and characterized a mouse model for the S47 variant in the background of humanized p53 (human *TP53* knock-in or Hupki)^20^ (Supplementary Fig. 1a). The Hupki platform has been used frequently to model TP53 SNPs and mutations in the mouse, and has proven a relevant and accurate model for genetic variation in human p53 (ref. ^22)^. We analyzed mouse embryonic fibroblasts (MEFs) derived from S47 mice, compared with P47 controls, as well as human lymphoblastoid cell lines from homozygous S47 and P47 individuals, for the level of iron using a calcein fluorescence assay. This assay showed evidence for increased iron in both human and murine S47 cells compared with P47. This iron is decreased upon deferoxamine (DFO) treatment (Fig. 1b). We previously reported that tissues from S47 mice are resistant to ferroptosis, and that these mice accumulate iron in the liver, particularly following hepatotoxic stress or aging^23^. Therefore, we next sought to analyze iron accumulation in the tissues of young mice S47 and P47 mice, using Prussian Blue staining. We observed that 6–8-week-old male S47 Hupki mice show evidence for increased iron accumulation in the spleen compared with P47 male mice, but not in other tissues (Fig. 1c; Supplementary Fig. 1b). Treatment of S47 mice with the iron chelator DFO depletes this excess iron from the spleen of S47 mice (Supplementary Fig. 1c).

We reasoned that the iron-containing cells in the spleen might be macrophages, as these are the main scavengers of iron in the body. To test this premise, we isolated splenocytes from P47 and S47 mice and used these to generate monocyte-derived macrophage cultures (MDMs) by virtue of their adherence to specialized culture plates. Consistent with our previous findings in S47 cells^20^ the S47 MDMs were highly resistant to death by the ferroptosis-inducing agents Erastin and RSL3 (Fig. 1d). These S47 MDMs also showed indicators of altered iron homeostasis, as indicated by a significant increase in the level of transferrin receptor (Tfrc); the high level Tfrc was a consequence of the iron accumulation, as this protein returned to near-basal levels following treatment of cells with the iron chelator DFO (Fig. 1e). These S47 MDMs showed increased Prussian Blue staining (Supplementary Fig. 1d), and splenocyte cultures from S47 mice showed increased iron content using inductively coupled plasma mass spectrometry (ICP-MS; Fig. 1f). In contrast, ICP-MS and Prussian Blue failed to reveal differences in iron content in sera or livers from these mice, and we only saw increased Tfrc in the livers of aged S47 mice (Fig. 1f; Supplementary Fig. 1e). Interestingly, we noted that the high-iron phenotype was markedly more dramatic in male S47 mice compared with female (compare Fig. 1f to Supplementary Fig. 1f). Therefore, we conducted many of the subsequent experiments, as noted, using male mice. We next assessed the impact of increased iron on the CBCs of P47 and S47 male mice. S47 male mice showed significantly higher red blood cell distribution width and monocyte counts, and significantly lower total leukocyte and lymphocyte counts (Fig. 1g), whereas other CBC parameters were not significantly changed (Supplementary Fig. 1h). These CBC alterations may be a consequence of the iron accumulation phenotype in S47 macrophages.

The S47 variant has been found only in natives of sub-Saharan Africa and their descendants. The allele frequency of the S47 variant is 1.2% in African Americans, and ~ 6% of Africans from sub-Saharan Africa. This allele has not yet been detected in Caucasian Americans (Fig. 1h, left panel). We sought to test the possibility that the S47 variant is associated with perturbed iron metabolism in African Americans. Approximately 1% of African Americans have high-iron phenotypes (elevated transferrin saturation, elevated serum ferritin levels, or both), but the underlying genetic basis for high-iron phenotypes in African Americans is multifactorial and incompletely understood^24^. To address the possibility that the S47 variant of p53 is implicated, we genotyped this SNP in DNA samples from the multicenter, multi-ethnic Hemochromatosis and Iron Overload Screening (HEIRS) Study. We genotyped 479 samples from African Americans who had elevated transferrin saturation with or without elevated serum ferritin (see details in Methods). As noted previously the frequency of the P47S allele is 1.2% in African-American populations^25^. In the present study, the frequency of P47S in African Americans from the HEIRS Study with high saturated transferrin and/or high serum ferritin (signs of iron overload) was 2.2% (OR 1.68, CI 1.07–2.65, *p* < 0.023) (Fig. 1h right panel). These data support the premise that there is a positive association of the S47 allele with high-iron phenotypes in African Americans.

### High iron causes severe bacterial pathogenesis in S47 cells

**Fig. 2:**
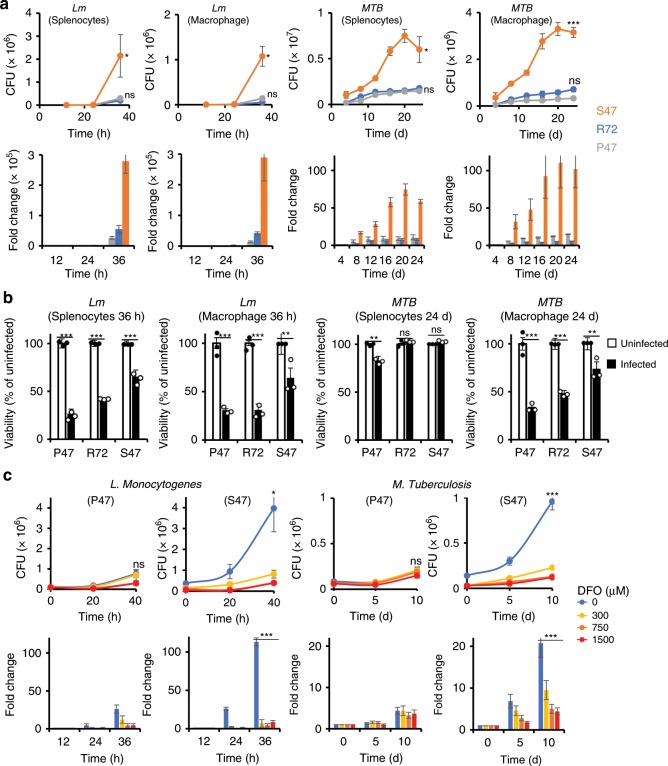
S47 cells show more intracellular bacterial growth owing to higher iron content. **a** Splenocyte and monocyte-derived macrophages (*n* = 3 biological replicates with three technical replicates) of P47, R72, and S47 mice were infected with Lm (Listeria) or MTB (M. tuberculosis), and bacterial viability was measured at the respective time points by CFU assay. Top panel—raw values, bottom panel fold change over 0 h/d post infection. Error bars represent means ± s.e.m. ***P < 0.001, *P < 0.05, ns—not significant; by unpaired Student’s *t* test, relative to P47 mice. **b** Splenocyte or macrophage viability with (black bars) or without (white bars) infection at the last time point after infection was measured by Resazurine cell viability assay (*n* = 3 biological replicates with three technical replicates). Error bars represent means ± s.e.m. ***P < 0.001, *P* < 0.01, *P < 0.05, ns —not significant; by unpaired Student’s *t* test, relative to the uninfected counterpart. **c** Monocyte-derived macrophages pretreated with PBS or the indicated DFO concentrations were infected with Lm or MTB and bacterial growth was measured by CFU at indicated time points (*n* = 3 biological replicates with three technical replicates). Top panel—raw values, bottom panel fold change over 0 h/d post infection. Error bars represent means ± s.e.m. ***P < 0.001, *P < 0.05, ns—not significant; by unpaired Student’s *t* test, relative to the DFO-treated counterpart. Source data are provided as a Source Data file.

We next infected mouse embryo fibroblasts (MEFs) from S47 and P47 mice with Lm or MTB; in addition, we analyzed MEFs from mice containing the R72 variant, which is the most common genetic variant of p53 in European Caucasians. We found that S47 MEFs infected with Lm or MTB support a higher level of bacterial growth compared with MEFs containing P47 or R72 (Supplementary Fig. 2a). This result was corroborated using total mouse splenocytes as well as isolated mouse macrophages from S47 mice. In both cases, S47 cells support a faster Lm or MTB growth and higher endpoint intracellular bacterial load compared with P47 and R72 mouse cells (Fig. 2a).

Activated macrophages phagocytose bacteria and kill them using reactive oxygen/nitrate species and solicit help from cytotoxic CD8 or NK cells through MHC1 or toll-like receptors (TLRs), respectively. If the infective pathogen is not killed, macrophages induce their own apoptosis, in order to contain the pathogen within the dying cell^26,27^. We observed higher viability of S47 mouse splenocytes and macrophages in the presence of Lm or MTB infection than in P47 or R72 mouse cells (Fig. 2b). This together with the high bacterial load suggests that the infected S47 macrophages are less effective at controlling Lm or MTB growth. We then treated P47 or S47 macrophages with DFO in order to chelate iron before infecting them with Lm or MTB. DFO treatment significantly reduced iron level in S47 macrophages (Supplementary Fig. 2b, c) along with Lm and MTB infection in S47 mouse macrophages while having little effect on bacterial growth in the P47 counterparts (Fig. 2c). DFO treatment did not have a significant effect on macrophage viability (Supplementary Fig. 2d).

### S47 mice are more susceptible to Listeria infection

**Fig. 3:**
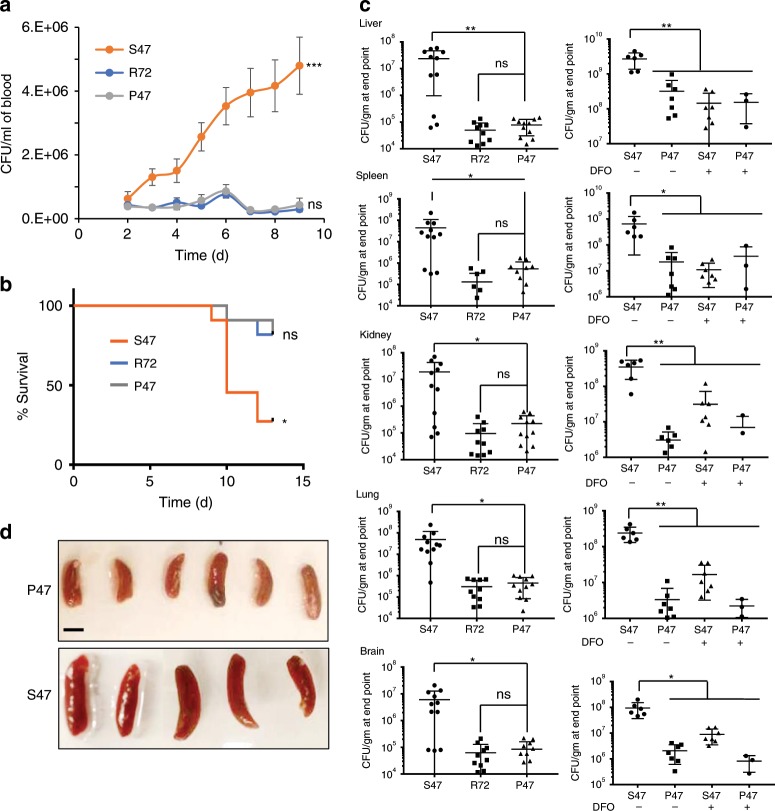
S47 mice are more susceptible to Listeria infection than WT mice. **a** S47 P47 or R72 mice were infected with Lm and monitored daily from day 2 post infection by CFU and **b** survival (*n* = 10 biological replicates). Error bars represent means ± s.e.m. ***P < 0.001, *P < 0.05, ns—not significant; by unpaired Student’s *t* test, relative to P47 mice. **c** Bacterial load in different organs at the time of death measured as CFU/gm (*n* = 7–10 biological replicates with three technical replicates). Right panel compares changes in bacterial load in P47 & S47 mice following DFO treatment. Error bars represent means ± s.e.m. *P* < 0.01, *P < 0.05, ns—not significant; by unpaired Student’s *t* test, relative to P47 mice. **d** Spleens of P47 and S47 mice at 13 days post infection. Bar = 1 cm. Source data are provided as a Source Data file.

To examine the in vivo significance of the S47 variant of p53 in infection clearance, we assessed the outcome of Lm infection in P47, S47, and R72 mice. S47 mice showed faster progression of Lm infection measured by daily blood CFU (Fig. 3a). Consistent with this, S47 mice showed significantly reduced survival, compared with P47 or R72 mice (Fig. 3b). To quantitate systemic infection, bacterial numbers in liver, spleen, kidney, lung and brain were measured. The S47 mice had an order of magnitude higher bacterial counts in every organ tested compared with P47 or R72 mice (Fig. 3c, left panels), leading to markedly higher splenic volumes in S47 mice (Fig. 3d). We next treated S47 mice with the iron chelator DFO for 3 weeks to deplete the iron stores from various organs, before inducing Lm infection. Marked changes in iron levels following DFO treatment were observed in the spleens (Supplementary Fig. 2e). Notably, this DFO treatment significantly reduced the systemic bacterial burden in S47 mice, to levels comparable to P47 counterparts (Fig. 3c, right panels).

### Proteomic comparison of P47 and S47 MDMs

**Fig. 4:**
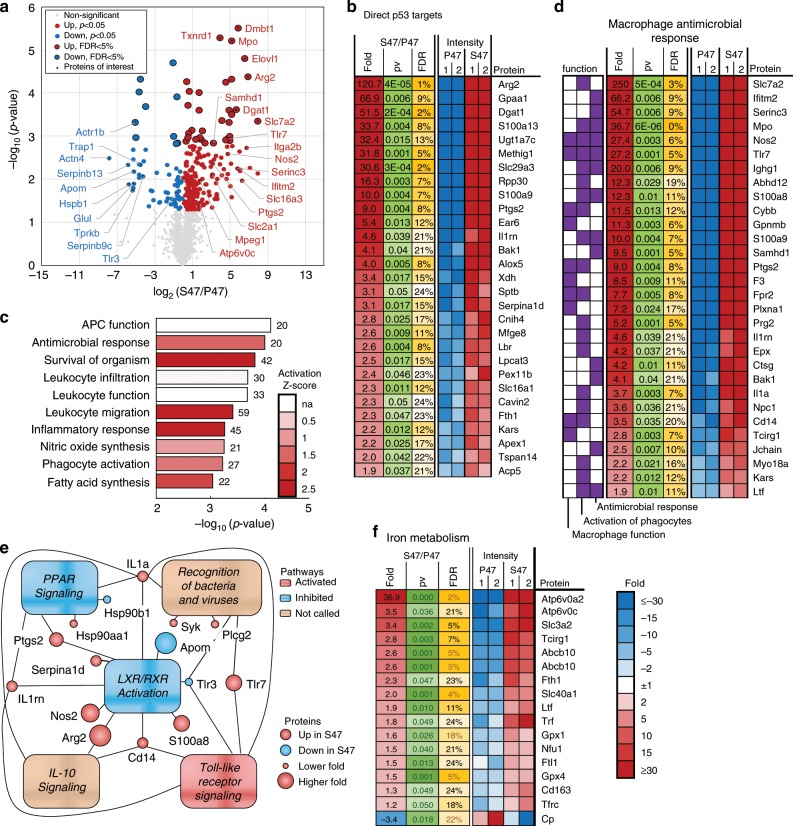
Proteomic analysis of P47 and S47 MDMs. **a** Volcano plot of proteins differentially expressed by P47 and S47 MDMs. Proteins of interest most of which have eightfold or higher changes are highlighted. **b** Known p53 target proteins with significantly different expression in P47 and S47 MDMs. *P* < 0.05 (unpaired *t* test) and FDR <25%. **c** Significantly enriched functions. Bars indicate the −log_10_(*p* value) with the number of proteins identified in each category next to the respective bar. The bars are color coded for their respective activation *z* scores. **d** Proteins important for macrophage innate immune response to bacterial infection are among the significantly enriched pathways in the proteomic screen *P* < 0.05 (unpaired *t* test) and FDR <25%. **e** Pathway analysis shows LXR/RXR activation and PPAR signaling pathways are suppressed in S47 MDMs. **f** Iron metabolism pathway proteins are significantly enriched in the proteomic screen *P* < 0.05 (unpaired *t* test) and FDR <25%. Source data are provided as a Source Data file.

To further dissect the mechanism for increased susceptibility of S47 mice to bacterial infections, we performed proteomics on MDMs isolated from the spleens of young (6–8 week old) P47 and S47 mice. We identified 286 proteins with levels significantly (*p* < 0.05 unpaired *t* test, FDR < 25%) changed in S47 MDMs. The top 28 proteins of interest, shown in the volcano plot, have distinct roles in the innate immune response (Fig. 4a; Supplementary Fig. 3a). Of the 286 proteins, 56 are known direct targets of *TP53* and 45 of those were significantly upregulated in S47 (Fig. 4b). Among the proteins most upregulated in S47 MDMs (Fig. 4a; Supplementary Fig. 3a) are Slc7a2 and Arg2, which are involved in the arginase pathway. Slc7a2 is a transmembrane arginine transporter and Arg2 is the mitochondrial arginase that converts arginine to urea and ornithine. The arginase pathway competitively suppresses the inducible nitric oxide synthase (iNOS) pathway which is responsible for killing intracellular and phagocytosed bacteria^28^. Other differentially expressed proteins include those involved in lipid and iron metabolism, innate immune signaling, metabolite transport, oxidative stress response and apoptosis (Fig. 4a, b; Supplementary Fig. 3a).

Upon analysis of the significantly changed proteins with Ingenuity Pathway Analysis (IPA) we found enrichment of ten major functions, nine of which were involved in the immune response to bacterial infection (Fig. 4c). This includes enrichment of proteins involved in macrophage function, activation of phagocytes and the antimicrobial response (Fig. 4d). These findings suggested that S47 MDMs may have a more anti-inflammatory phenotype. In addition, we observed significant change in the levels of 6 proteins involved in ferroptosis, including Glul and Trap1, which were downregulated at least 20-fold in S47 MDMs (Supplementary Fig. 3b). This may play a role in the resistance of these cells to ferroptosis (Fig. 1d)^11^. IPA also revealed significant enrichment of proteins from 19 pathways, including those responsible for bacterial recognition, phagosome formation and signaling by IL10, IL7, eicosanoid (prostaglandin), NF-κB, TREM1, and toll-like receptor (TLR) signaling (Supplementary Fig. 3c and 3e). The liver X receptor/retinoid X receptor (LXR/RXR) and the peroxisome proliferator-activated receptor (PPAR) pathways were the only two pathways predicted to be suppressed (negative activation state *Z* scores) in the S47 MDMs. Both pathways play an important role in regulation of proinflammatory macrophage response and macrophage associated killing of bacteria^29,30^ (Fig. 4e). Not surprisingly, IPA also revealed enrichment of proteins involved in the iron metabolism pathway in S47 cells (Fig. 4f).

### S47 mouse macrophages show anti-inflammatory polarization

**Fig. 5:**
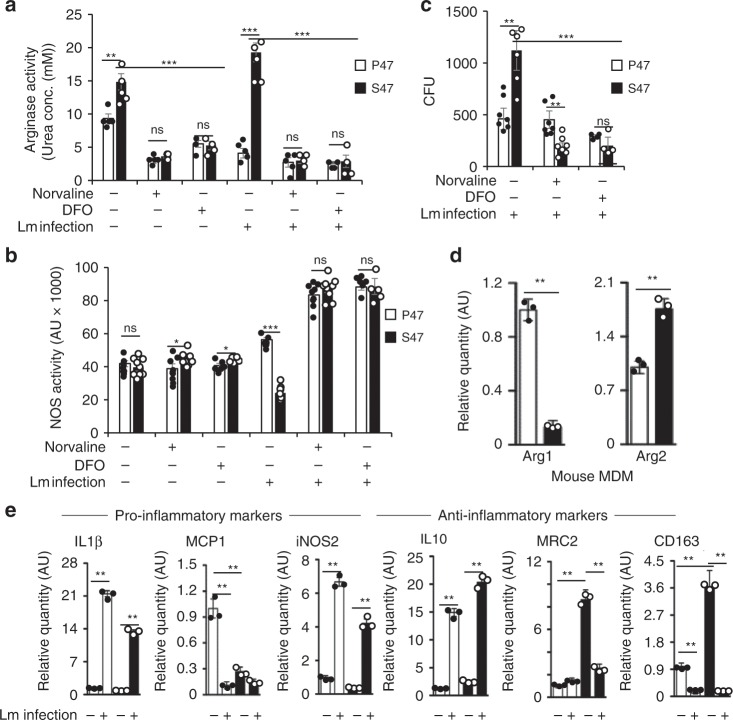
S47 mouse macrophages exhibit anti-inflammatory response to Lm infection. **a** Arginase activity, **b** NOS activity, and **c** bacterial CFU, measured in P47 (white) and S47 (black) under indicated conditions (*n* = 5 biological replicates and three technical replicates). Error bars represent means ± s.e.m. ***P < 0.001, **P < 0.01, *P < 0.05, ns—not significant; by unpaired Student’s *t* test, relative to P47 mice or to S47 mice where indicated. **d** Relative expression data of Arg1 and Arg2 by qRT-PCR analyses of male P47 (white) and S47 (black) mouse MDMs. Error bars represent means ± s.e.m. *n* = 3, **P < 0.01, by unpaired Student’s *t* test, relative to the uninfected counterpart or to P47 mice where indicated. **e** mRNA levels of pro- and anti-inflammatory markers in P47 (white) and S47 (black) mouse macrophages measured by qRT-PCR (*n* = 3 biological replicates and three technical replicates). Error bars represent means ± s.e.m. **P < 0.01, by unpaired Student’s *t* test, relative to the uninfected counterpart or to P47 mice where indicated. Source data are provided as a Source Data file.

High arginase and low NOS activity are associated with anti-inflammatory macrophage responses^28,31^. Recent reports have linked arginase activity to poor outcomes in bacterial infections^32,33^ and previous reports have shown a link between iron levels and arginase activity^34,35^. We compared the arginase and NOS activity in mouse macrophages isolated from P47 and S47 males. At steady state, S47 macrophages have significantly higher arginase activity than P47 macrophages (Fig. 5a). This difference is more notable upon Lm infection, as S47 macrophages increase their arginase activity while their P47 counterparts decrease their arginase activity (Fig. 5a). Both P47 and S47 macrophages show similar NOS activity at steady state but the S47 NOS activity drops significantly upon Lm infection (Fig. 5b). Notably, the difference in P47 and S47 arginase and NOS activities is completely abrogated by treating macrophages with the arginase inhibitor Norvaline or the iron chelator DFO (Fig. 5a, b). As expected, Lm bacterial load in S47 macrophages is significantly higher than in P47 macrophages. However, Norvaline- or DFO-treated S47 macrophages show markedly improved ability to kill bacteria, to levels comparable or better than their P47 counterparts (decreased CFU, Fig. 5c). This difference is most likely attributable to the increase in NOS activity by Norvaline or DFO treatment. We next used Quantitative RT-PCR (Q-PCR) and showed that S47 MDMs have a higher level of Arg2 mRNA and lower Arg1 expression compared with P47 counterparts (Fig. 5d). Q-PCR of RNA from infected or uninfected macrophages also showed higher levels of proinflammatory (M1) markers IL1β, MCP1 and iNOS2 in P47 macrophages, but conversely higher levels of anti-inflammatory (M2) markers IL10, MRC2, and CD163 in S47 macrophages (Fig. 5e). Again, we found that these differences in pro/anti-inflammatory markers to be most significant in macrophages isolated from male mice but much less-pronounced in macrophages isolated from female mice (Supplementary Fig. 4a).

### LXR agonists improve Lm killing by S47 mouse macrophages

**Fig. 6:**
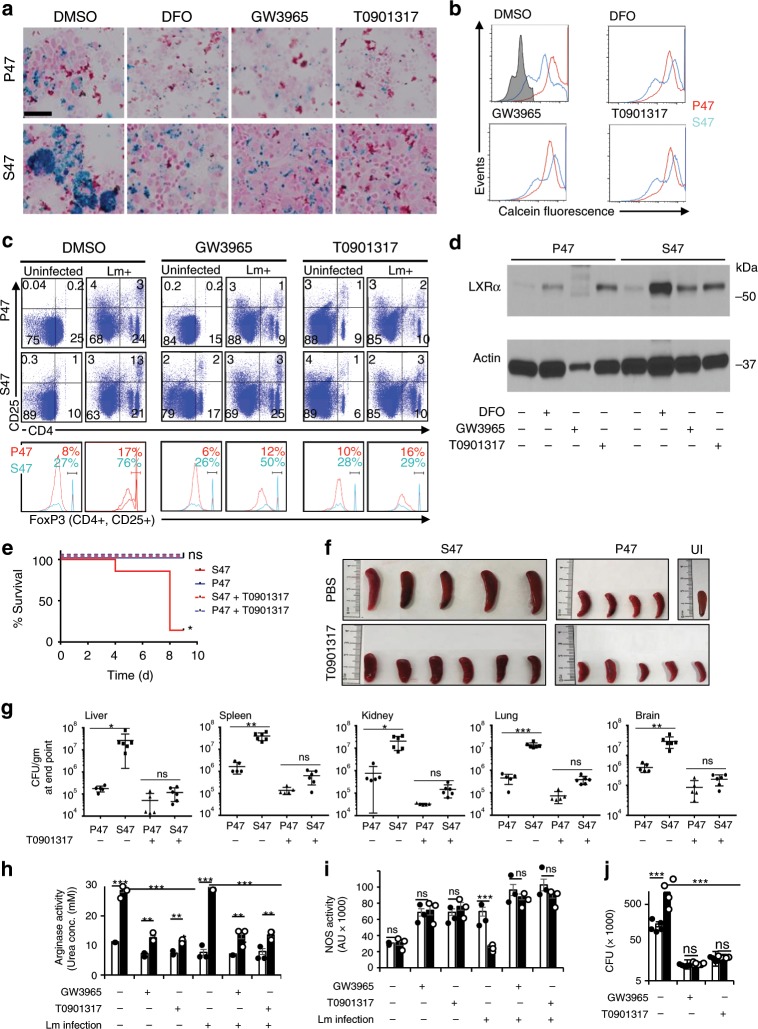
LXR agonists lower iron content, reverse anti-inflammatory response, and improve Lm killing in S47 mouse macrophages. **a** Iron accumulation in P47 and S47 MDMs under different treatment conditions by Prussian blue staining. (*n* = 10 biological replicates, 3–4 fields per sample), Bars, 50 μm. **b** Iron levels in P47 and S47 MDMs under different treatment conditions measured by loss of calcein fluorescence. Gray histogram = unstained control. (representative from 10 biological replicates). **c** P47 and S47 mouse splenocytes pretreated with DMSO, GW3965, or T0901317 analyzed for CD4 + , CD25 + and FoxP3-Hi Treg at baseline or in the presence of Lm infection. **d** Relative protein levels in P47 and S47 mouse MDMs measured by immunoblot after 24 h of indicated treatments. **e** S47 & P47 mice infected with Lm are treated with PBS or LXR agonist T0901317 and monitored daily from day 2 post infection for survival (*n* = 5–6 biological replicates) **f** Spleens of P47 and S47 mice at 9 days post infection. scale = 4 cm. **g** Bacterial load in different organs at the experimental endpoint measured as CFU/gm (*n* = 5–6 biological replicates with three technical replicates), compares changes in bacterial load in P47 & S47 mice following T0901317 treatment. Error bars represent means ± s.e.m. *P* < 0.01, *P < 0.05, ns—not significant; by unpaired Student’s *t* test, relative to P47 mice. **h** Arginase activity, **i** NOS activity, and **j** Bacterial CFU, measured in P47 (white) and S47 (black) under indicated conditions (*n* = 5 biological and three technical replicates). Error bars represent means ± s.e.m. ***P < 0.001, **P < 0.01, ns—not significant; by unpaired Student’s *t* test, relative to P47 mice or to S47 mice where indicated. Source data are provided as a Source Data file.

Pathway analysis from our proteomics data show inhibition of liver X receptor (LXR) pathway in S47 macrophages (Fig. 4e; Supplementary Fig. 3c). LXR activity is important for macrophage inflammatory activity^36,37^ and for iron export-induced proinflammatory (M1) responses in macrophages^38^. It is also important for the induction of protective antibacterial immune response against Lm and MTB^29,39,40^. We hypothesized that LXR activation by the chemical agonists GW3965 and T0901317 might induce a proinflammatory response and aid in bacterial killing by S47 macrophages. We also reasoned that it might reduce iron levels as well. Indeed, iron levels in S47 mouse MDMs decreased following treatment with the LXR agonists GW3965 and T0901317 (Fig. 6a, b). Ex vivo S47 mouse splenocytes infected with Lm show greater numbers of CD25+, CD4+, FoxP3-Hi Tregs, 24 h post infection. These Treg numbers, however, are suppressed upon treatment with LXR agonists (Fig. 6c). As expected, LXR agonist treatments increased the expression of LXRα in MDMs, and the response was increased in S47 MDMs (Fig. 6d). S47 mice treated with LXR agonist T0901317 showed significant improvement in survival post Lm infection (Fig. 6e). LXR activation in S47 mice rescued splenic enlargement owing to listeria infection (Fig. 6f) and led to a significant decrease in bacterial load in liver, spleen, kidney, lung, and brain (Fig. 6g). LXR agonists also led to suppressed arginase activity, improved NOS activity and NO release in S47 MDMs, especially after Lm infection (Fig. 6h, i). This proinflammatory response induced by LXR activation is corroborated by improved killing of Lm in infected S47 mouse MDMs (Fig. 6j).

We next replicated these findings in ex vivo models of *Salmonella typhimurium* and *Yersinia pestis* infections. In both cases, S47 macrophages support faster bacterial growth and higher end point intracellular bacterial load compared with P47 cells (Supplementary Fig. 4b, c). Treatment with DFO or the LXR agonist T0901317, significantly reduced *Salmonella* and *Yersinia* infection in S47 mouse macrophages while having little effect on bacterial growth in the P47 counterparts. We observed higher viability of S47 mouse macrophages in the presence of *Salmonella* or *Yersinia* infection than in P47 mouse cells, which suggests that the S47 macrophages infected with these bacteria are less effective at apoptosis. Finally, the higher viability of S47 MDMs was rescued by DFO or T0901317 treatment (Supplementary Fig. 4d).

We next further explored the inflammatory response in P47 and S47 splenic MDMs following infection with Lm. At steady state conditions P47 MDMs released significantly higher levels of proinflammatory cytokines MCP5, MIP1α, MIP1β, MIP2, RANTES, SDF1, and IL17 into the culture media while S47 MDMs produced significantly higher IL10 (Supplementary Fig. 5). In the presence of active Lm infection the P47 MDMs show a more potent proinflammatory response with significantly higher levels of IL1α, IL1β, and IL17, whereas their S47 counterparts show a more pronounced anti-inflammatory response with high IL10 levels. Treating the MDMs with DFO or the LXR agonist GW3965 prior to Lm infection abrogates the anti-inflammatory response in S47 MDMs by lowering IL10 and increasing IL1α, IL1β, and IL17 secretion (Supplementary Fig. 5a, b). These combined data support an iron- and LXR-regulated difference in inflammatory response between P47 and S47 MDMs; these differences likely explain the improved response to S47 MDMs to Lm and MTB following DFO treatment (Figs. 2d, 3c).

### S47 mice exhibit an anti-inflammatory response to hemozoin

As the S47 variant of p53 is most prevalent in malaria-endemic regions of Africa, we next sought to test the hypothesis that this variant might confer a survival advantage in response to malaria infection. In this case we assessed the response of P47 and S47 mice to hemozoin, as several lines of evidence indicate that this malarial pigment is principally responsible for the severity of malarial immunopathology; as one example, the likelihood of developing cerebral malaria increases with the amount of hemozoin released into the circulation during an active infection^41–44^. P47 and S47 mice were injected with hemozoin and compared for their peritoneal and splenic immune cell profile and peritoneal cytokine profile. P47 mice showed higher total (Gr-1+) and activated (Gr-1+, CD11b+) neutrophils in their peritoneum compared with S47 mice. Conversely, S47 mice showed a greater percentage of (F4/80+) macrophages in the peritoneum, but these macrophages also expressed higher levels of the M2 (anti-inflammatory) marker Egr2 (Supplementary Fig 6a)^45^. In response to hemozoin, the S47 peritoneal wash showed greater numbers of regulatory T cells (Tregs, CD25+, FoxP3+) and fewer activated CD4 and CD8 T cells (CD69+), indicative of an impaired inflammatory response (Supplementary Fig. 6b). Similar results were seen with isolated P47 and S47 mouse splenocytes (Supplementary Fig. 4e, f). Using a dot-blot array, we measured the intra-peritoneal cytokines released by P47 and S47 in response to the injected hemozoin (Supplementary Fig. 6c). P47 mice show significantly higher levels of proinflammatory (pyrogenic) cytokines IL1, IL6, IL12, IL17, MCP1, MCP5, MIP1α, MIP1β, and TNFα, whereas S47 mice show significantly higher levels of anti-inflammatory IL10 (Supplementary Fig. 6d). Consistent with these findings, we found that incubation of P47 MDMs with hemozoin produced significantly higher CXCL1 and TNFα, whereas S47 MDMs produce markedly higher levels of IL10 (Supplementary Fig. 5). These combined findings indicate that the malarial toxin hemozoin in P47 macrophages induces a marked proinflammatory response, whereas S47 macrophages respond with an increased anti-inflammatory response.

## Discussion

We previously showed that cells containing the S47 variant of *TP53* display a defect in ferroptosis; this defect is caused by a decreased ability of this variant to regulate p53 target genes involved in ferroptosis, including GLS2 and Slc7a11 (ref. ^20)^. Here-in we show that the ferroptotic defect in cells containing the S47 variant of p53 is accompanied by an iron accumulation phenotype, predominantly in the macrophages of mice. We show that the iron accumulation in S47 macrophages renders them more conducive to bacterial growth, and that S47 mice are more susceptible to bacterial infection. Iron is one of the most-essential nutrients required for bacterial growth, and our macrophages have evolved a system of sequestering iron away from phagocytosed bacteria using the NRAMP1/2 iron transporter^17,18^. Patients with iron overload from hemochromatosis are more susceptible to infections with *Yersinia, Listeria, Vibrio,* and *Mycobacterium*^46,47^. For this reason, iron supplementation is avoided in patients with active or resistant infections. Since 95% of recycled iron in the body passes through macrophages, it is logical that these cells show the most prominent difference in iron levels when comparing S47 and P47 tissues. Chelating iron from S47 macrophages lowered their bacterial burden and S47 mice showed significantly lower bacterial dissemination in their organs with DFO treatment.

We conducted a proteomic comparison of P47 and S47 mouse MDMs in order to understand the mechanistic differences induced by the S47 SNP in macrophages. Many of the significantly changed proteins fell into pathways directly responsible for macrophage activation and function, antimicrobial and inflammatory responses, leukocyte infiltration, and bacterial killing by NO synthesis. Although we focused this study on the antimicrobial immune response pathways, analysis of our proteomics data also revealed disparities in pathways responsible for protein synthesis, molecular transport, cell cycle, cardiovascular disease, amino acid/lipid/energy metabolism, and cell signaling. This suggests that iron could be serving a more-extensive role besides being a bacterial nutrient^48^. Two of the top 10 proteins upregulated in S47 MDMs were the arginine importer Slc7a2 and the mitochondrial arginase Arg2. The most important mechanism of bacterial killing by phagocytic macrophages involves iNOS activation and release of highly oxidative NO into the phagolysosome. Although human monocytes do not produce large amounts of NO compared with mouse monocytes, in the past several years accumulating data have suggested that iNOS expression has an emerging role in human immune and chronic inflammatory disease^49^. As the iNOS pathway uses arginine as a substrate, its activity is controlled by the arginase pathway, which also uses arginine to produce ornithine, a precursor for polyamines. Arginase activity in macrophages is important for resolution of inflammation, wound healing, and anti-inflammatory immune response^28,31,50^. High arginase activity has also been implicated in poor prognosis of bacterial infections^32,33^. Previous studies have linked iron accumulation to arginase activation and anti-inflammatory (M2) polarization of macrophages in vitro^34,48^. Consistent with this, we show higher arginase activity in healthy S47 MDMs. Upon Lm infection, while P47 MDMs lower their arginase activity and increase their NO release, S47 MDMs react the opposite way. The result of this anti-inflammatory response reflects in the high bacterial burden in Lm-infected S47 MDMs. The phenotype is completely reversed upon arginase inhibition by norvaline or iron chelation by DFO and the S47 bacterial burden decreases significantly.

Our proteomic analysis indicated that the LXR pathway was inhibited in the S47 MDMs. LXRs are nuclear receptors for endogenous oxysterols and regulate glucose, cholesterol, and fatty-acid homeostasis. The expression of the isoform LXRα is restricted to macrophages and a few other tissues while LXRβ is expressed more ubiquitously. LXR activation is important for inducing an inflammatory response in macrophages^36,37^, export of iron deposits from macrophages^38^ and for macrophage mediated killing of bacteria^39,40,51^. Upon further dissection we discovered that the LXR agonists GW3965 and T0901317 reversed the iron accumulation in S47 MDMs and abrogated the in vitro expansion of CD4+ CD25+ FoxP3-Hi Tregs seen as a response of S47 splenocytes to Lm infection. We also noted a decrease in secretion of IL10 and an increase in secretion of proinflammatory cytokines and chemokines by S47 MDMs. Activation by the LXR agonist drugs suppressed the high arginase activity in S47 MDMs and increased NO release in response to Lm infection. Owing to this reversal of S47 MDM anti-inflammatory response, the bacterial load in infected S47 MDMs decreased significantly.

The genetic basis for primary iron overload in African Americans is not understood. Homozygosity for *HFE* p.C282Y and p.C282Y/p.H63D compound heterozygosity account for most hemochromatosis cases in European Americans, but these alleles are uncommon in African Americans^52–54^. We show that the S47 allele is over-represented in African Americans with elevated transferrin saturation and serum ferritin levels in the HEIRS Study. The majority of African-American HEIRS Study participants with S47 we detected were heterozygotes. It is unknown whether S47 homozygotes have more severe high-iron phenotypes than S47 heterozygotes. Primary iron overload is typically more pronounced in men than women, although the reason(s) for male bias are incompletely understood. In our mouse model, iron accumulation was more severe in males. It has been postulated that the regulation of hepcidin by female hormones may decrease primary iron overload in women^55^. It is of interest that the exacerbation of iron accumulation in males was recapitulated in our mouse model.

The S47 SNP is predominantly found in people living in Sub-Saharan western Africa, where malaria is endemic. In an inbred mouse model, we find that this SNP confers an apparent advantage to malaria immunopathology. Similar studies in humans need to be performed, to assess a correlation between humans with S47 and malaria pathology; such studies will need to control for the known protection afforded by mutations in the globin genes. Consistent with our findings, others have found that p53 status and activity can alter the sensitivity to liver stage malaria^56,57^. During human or murine malaria, phagocytes (macrophages and to lesser extent neutrophils) play a crucial role by engulfing free parasites and *Plasmodium*-infected RBC and eliminating them^58,59^. Macrophages are also the main source of cytokines IL1, IL6, IL12, and TNFα in malarial infection, which greatly exacerbates the inflammatory response^60,61^. The severity of malaria infection and its progress to the cerebral stage is linked to the levels of the malarial pigment hemozoin released into circulation, which ultimately ends up in the phagocytic leukocytes (neutrophils, monocytes, and macrophages)^42–44^. The impact of iron on malaria risk has been controversial; some have argued against iron supplementation in malaria-endemic regions for fears that this would exacerbate malaria infection and pathology, whereas others have found that iron supplementation defers lethal malaria infection in a mouse model^62^. In support of the latter premise, some hemochromatosis alleles have been found to protect against severe malaria disease in mouse models^63,64^.

Our studies show that S47 mice injected IP with hemozoin have significantly lower levels of activated neutrophils in the peritoneum compared with P47 mice. More S47 monocytes/macrophages infiltrating the peritoneum have the anti-inflammatory (M2) marker Egr2, than their P47 counterparts. We also notice significantly lower levels of proinflammatory cytokines in peritoneal washes from hemozoin-injected S47 mice. However, the levels of IL10, the anti-inflammatory cytokine, are significantly higher in S47 mice. This could explain the higher levels of Tregs in the peritoneum infiltrate of hemozoin-injected S47 mice. These findings suggest that the anti-inflammatory phenotype of the S47 SNP might limit the severity of malarial immunopathology and help people survive in malaria-endemic regions where the entomological inoculation rates can be as high as 400 infectious bites per person per year^65^. We believe mechanistic knowledge from studying the S47 SNP will not only be a stepping stone in the field of personalized medicine to address health disparities arising from such polymorphisms but will also help understand the link between bacterial infections and other forms of iron overload disorders.

## Methods

### Bacteria

*L. monocytogenes* (Lm) 10403 s strain^66^, MTB H37ra, *Salmonella enterica typhimurium* (LT2–SL7207) and *Yersinia pestis* (KIM 10+) (BEI Resources) were used. Lm and MTB were grown at 37 °C in 2.5% brain heart infusion (BHI) or Middlebrook 7H10 with oleic albumin dextrose catalase (OADC) media, respectively. LB and TSA + 5% blood agar were used to culture *Salmonella* and *Yersinia,* respectively.

### Animal models

All studies were carried out in accordance with the recommendations in the Guide for the Care and Use of Laboratory Animals of the National Institutes of Health (NIH). All protocols were approved by The Wistar Institute and the Perelman School of Medicine at the University of Pennsylvania, Institutional Animal Care and Use Committee (IACUC). We have complied with all relevant ethical regulations for animal testing and research. Wild-type (WT) and S47-humanized *TP53* knock-in (Hupki) mice were generated by Dr. Maureen Murphy (The Wistar Institute, Philadelphia, PA)^20^. All mice were backcrossed to C57bBl/6 for >10 generations, and as much as feasible, sibling littermates were used for these analyses. Mice were housed in plastic cages with ad libitum diet and maintained with a 12-hr light/dark cycle at 22 °C. Male mice were used for all studies once data revealed modest differences in iron levels between WT and S47 female mice. Controls and experimental groups were age and genotype-matched non-littermates. Young mice are considered 6–12 weeks of age, whereas old mice are all 52 weeks or older. For CBC analysis, WT and S47 mice were killed followed by cardiac puncture to obtain blood for serum and hematological analysis. Spleens were harvested for ICP-MS and ex vivo infection studies.

### Cell lines, MDM cultures

The Coriell institute maintains human lymphoblastoid cell lines (GM18870 and GM18871) generated from B lymphocytes isolated by the International HAPMAP project. The B-lymphocytes were isolated from members of a Yoruba family carrying S47 and P47 homozygous SNP from Ibadan, Nigeria. For MDM cultures, splenocytes were isolated from P47 or S47 mouse spleens and kept in Primaria plastic plates for the monocytes to adhere and differentiate to macrophages.

### Human samples

We analyzed the *TP53* SNP rs1800371 in 479 African-American samples, both men and women who were HEIRS Study participants ≥25 years of age. Samples were obtained from participants after they provided informed consent. All participants had elevated transferrin saturation (>50%); in approximately one quarter of the cases, participants also had serum ferritin values >300 µg/mL. DNA samples or buffy coat punches were obtained from the HEIRS Study, part of the BioLINCC repository of the National Heart Lung and Blood Institute. For the Buffy coat punches, DNA was extracted using the QIAamp DNA Mini prep kit (Qiagen, Cat # 51306) as per vendor instructions. DNA was quantitated using NanoDrop 1000 (ThermoFisher Scientific) and 10–20 ng of DNA was used as input for Allelic Discrimination Endpoint PCR on the 7900HT Fast Real-Time PCR system (Thermo Fisher Scientific). PCR was done in triplicate wells using premade TaqMan SNP Genotyping assay primer/probe set (Cat # 4351379, Assay ID: C_8727792_10, RS number: rs1800371; context sequence aligns with human p53 exon 4 coding sequence) and TaqMan Universal PCR Master Mix No AmpErase UNG (Thermo Fisher, Cat # 4324018). Control samples: P47 p53 homozygous, S47 p53 mutant homozygous and a 50/50 mixture of both samples were assayed in each Endpoint PCR run to determine the genotype of each sample. To control for *HFE* mutation status such that homozygotes and compound heterozygotes for the p.C282Y and p.H63D could be excluded, we performed TaqMan genotyping using primer sets for rs1800562 and rs1799945, using control DNA from the Coriell Institute from known affected individuals NA11831 and HG01136, respectively. The human study on de-identified human samples was approved by the Wistar Institutional Review Board (Federal wide Assurance #00005453). We have complied with all relevant ethical regulations and informed consents were obtained by the HEIRS study group.

### Primers used

The following primers were used in this study: primers for CD163 (fwd: ATGGGTGGACACAGAATGGT and rev: AGCTCACAGCCACAACAAAG), primers for IL1β (fwd: TGTGAAATGCCACCTTTTGA and rev: GGTCAAAGGTTTGGAAGCAG), primers for IL10 (fwd: AGAGAAGCATGGCCCAGAAA and rev: ACACCTTGGTCTTGGAGCTT), primers for Mcp1/Ccl2 (fwd: CCTGCTGTTCACAGTTGCC and Rev: ATTGGGATCATCTTGCTGGT), primers for Mrc2 (fwd: GATCCACGAGCAGACCTACA and rev: TTCTCCTCACCTGGGTTGTC), and primers for Nos2 (fwd: CCTTGTTCAGCTACGCCTTC and rev: CTTCAGAGTCTGCCCATTGC).

### Mouse infection and inflammation studies

In certain experiments mouse infection was induced by injecting 10^6^ Lm per mouse intraperitoneally (i.p.) in 200 μl DPBS. Where mentioned, deferoxamine mesylate (DFO, 50 mg/kg per mouse) in 1× Dulbecco's phosphate-buffered saline (DPBS) was injected i.p. once a day once a day for three weeks, before inducing the infection or killing them for tissue analysis, as indicated. Blood from infected mice was collected daily using tail snips and analyzed for bacteremia by CFU. Following death from infection or euthanasia at the end of the experiment, spleen, liver, lung, brain and kidneys were harvested, sectioned, and studied for bacterial CFU, immunohistochemistry (IHC), iron staining, or flow cytometry as indicated. The immune responses of P and S47 mice to malarial pigment hemozoin were studied by injecting 2.5 mg hemozoin per mouse, intraperitoneally. After 8–12 h mice were killed and their peritoneal washes and spleens collected. Cells from the peritoneal wash were analyzed by FACS and the supernatants analyzed for their cytokine profiles.

### Isolation of splenocytes and MDMs

Splenocytes were isolated by crushing spleens of healthy age-matched mice in 12-well plastic tissue culture plates using a 5 ml syringe. RBCs were lysed in RBC lysis (ACK) buffer at 37°C and for 1 min. Cells were washed 3–5 times with MACS buffer at 4 °C. Splenocytes were then either frozen and sent for ICP-MS studies or plated on 6, 12, 24, or 96 well Primaria plates (Fischer Scientific, Cat #08-772) in 10% FBS RPMI medium supplemented with 100 U/ml penicillin G and 100 mg/ml streptomycin sulfate, 6 mm HEPES, 1.6 mm l-glutamine, 50 mm β-mercaptoethanol. Within 3–4 days monocytes adhere to the surface and transform to MDMs^66^. The suspension cells and antibiotics are washed away where indicated and the MDMs (kept in antibiotic free culture medium) are used for ex vivo infection models, Prussian blue staining for iron content, used for arginase/NOS activity assays, hemozoin assay or lysed for mRNA profiling, proteomics or western blots. Splenocytes for ICP-MS and ex vivo infection studies were obtained by harvesting spleens of sacrificed healthy mice.

### Ex vivo infection in MDMs

Where indicated the MDMs are pretreated at 37°C with LXR agonists 10 μm GW3695 or T0901317 for 4 hours, or DFO (300, 750, 1500 μm) overnight or Norvaline (20 mm) overnight. Lm and MTB ex vivo infections in MDMs are induced at a (multiplicity of infection) MOI of 1:1 and 1:10 respectively. For *Yersinia* and *Salmonella* an MOI of 1:0.1 was used. Infected MDMs were lysed in distilled water at different time points (12, 24, and 36 h for Lm and 24, 48, and 72 h for MTB) and the lysates used for CFU analysis^66^. At the last time points media supernatants were collected for cytokine profiling. In certain experiments MDMs were also used to measure arginase and iNOS activity.

### CFU analysis

Lysates from mouse blood, tissues or ex vivo MDMs were serially diluted and 50 μl plated on bacterial culture plates. BHI agar plates for Lm and Middlebrook 7H10 agar plates with OADC growth supplement for MTB were incubated at 37 °C. Lm *Yersinia* and *Salmonella* colonies were counted after overnight incubation and MTB colonies after a 20-day incubation. The CFU were normalized per ml for blood or per gram weight for tissues. All experiments were replicated in at least three independent experiments with multiple technical replicates in each experiment.

### CBC analysis

Blood from each mouse was split into two tubes: half was placed in a Serum Separator Microtainer (BD, cat. # 365967), whereas the other half was placed in a MiniCollect Tube (0.5 mL, K2E K2EDTA) (Greiner Bio-One, cat. # 450480) and whole blood was submitted to the Ryan Veterinary Hospital Clinical Pathology Laboratory (University of Pennsylvania) for complete blood count (CBC) analysis using a ProCyte DX Hematology Analyzer (IDEXX Laboratories, Westbrook, ME).

### ICP-MS analysis, calcein assays

Serum, splenocytes, and isolated tissues were sent to the toxicology lab of the Pennsylvania Animal Diagnostic Laboratory System (PADLS) for ICP-MS analysis of iron levels. The tissue samples were dried overnight in an oven maintained at 70 °C and then weighed into Teflon PFA vials (Savillex, Minnetonka, MN). The dried tissue samples were digested overnight with 20 times the amount (weight/volume) of 70% nitric acid. Fluid and splenocyte samples were weighed and then digested overnight with twice the amount (weight/volume) of concentrated nitric acid. Digestion of all samples was done in the oven set at 70 °C. A 0.1 mL portion of digested tissue sample or 0.15 mL of digested fluid or splenocyte sample was mixed with 0.05 mL of 2 ppm internal standard containing Ge (geranium), In (indium), Tb (terbium), and Y (yttrium). The mixture was diluted with deionized water to a final volume of 5 mL for analysis. The concentration of iron in the submitted sample was measured using a calibration curve of aqueous standards prepared at four different iron concentrations. The samples were analyzed for iron using a Nexion 300D ICP-MS (Perkin Elmer, Shelton, CT) at the PADLS New Bolton Center Toxicology Laboratory, University of Pennsylvania School of Veterinary Medicine, Kennett Square, PA. The analytical standards were purchased from SCP (Champlain, NY) and trace metal grade nitric acid was acquired from Fisher Scientific (Pittsburg, PA). All dilutions were done using an in-house deionized water (≥18 MΩ) obtained from a water purification system (EMD Millipore, Billerica, MA). The performance of the instrument and the accuracy of the results was monitored by analyzing a reagent blank and a matrix matched reference material, with known values of iron, with each batch of samples. Results were reported in ppm, on a dry weight basis for tissues and wet weight basis for fluids and splenocyte samples. For the calcein assays, 3 × 10^5^ cells per sample were incubated with 500 nm of Calcein AM (Thermo Fisher C1430) for 30 mins at 37 °C. After a PBS wash, cells were analyzed by flow cytometry using a BD FACSCelesta, applying a 488 nm beam laser and analyzing at least 10,000 events per sample.

### RNA isolation and quantitative RT-PCR

Total RNA was extracted using the Direct-zol RNA Kit (Zymo Research, Tustin, CA), according to manufacturer's instructions. Total RNA samples were reverse-transcribed using the SuperScript III First-Strand Synthesis System, and the resulting cDNA was used with Fast SYBR Green Master Mix and primer sets, as listed. Quantitative RT-PCR was performed using the QuantStudio 7 Flex Real-Time PCR System (ThermoFisher Scientific). Ct values for the genes of interest were normalized to the invariant control gene p38. Gene expression data are expressed as fold change relative to untreated or vehicle-treated P47 controls. All experiments were replicated in at least three independent experiments with technical replicates in each experiment, as indicated.

### Tissue staining and IHC

Tissues were harvested and fixed in Formalde-Fresh Solution overnight at 4 °C, washed with 1 × PBS and transferred to 70% ethanol before paraffin embedding and sectioning. Tissue embedding and sectioning were performed by the Molecular Pathology & Imaging Core of the Penn Center for Molecular Studies in Digestive and Liver Diseases (Perelman School of Medicine, University of Pennsylvania) and by the Histotechnology Facility (The Wistar Institute). For IHC studies, tissue sections were deparaffinized in xylene, rehydrated in ethanol (100%–95%–80%–70%) and then distilled water. For Sirius Red staining, the tissue sections were immersed in Picro-sirius Red solution (0.5 g of Direct Red 80/Sirius Red in 500 ml of Picric acid solution) for 1 h at room temperature, washed three times with acidified water (5 ml of glacial acetic acid diluted in 1 liter of distilled water), dehydrated in ethanol, cleared in xylene and mounted using Cytoseal 60. For Prussian Blue staining, tissue sections were deparaffinized in xylene, rehydrated in ethanol (100%–95%–80%–70%) and then distilled water. Prussian blue iron stain kit (Polysciences, Inc) was used to stain the tissue. Tissue sections were subsequently immersed in Prussian Blue Staining Solution (equal parts of 20% hydrochloric acid and 10% potassium ferrocyanide solution) for 0.5–1 h at 37 °C, washed with distilled water and counterstained with Nuclear fast red for 5 min at room temperature. The slides were then dehydrated in ethanol, cleared in xylene and mounted using Cytoseal 60. For F4/80 staining, tissue sections were subjected to Proteinase K antigen retrieval protocol at room temperature. After quenching endogenous peroxidase activity with 3% hydrogen peroxide, tissue sections were treated with avidin/biotin blocking reagents and StartingBlock T20 (PBS) Blocking Buffer, according to manufacturer instructions. Tissue sections were subsequently incubated with vendor recommended dilutions of primary antibody overnight at 4 °C, washed next day with 1× PBS and incubated with biotinylated secondary antibody for 30 min at 37 °C. Antibody complexes were then detected using the Vectastain Elite ABC HRP Kit and DAB peroxidase (HRP) Substrate Kit, according to manufacturer instructions. Tissue sections were then dehydrated in ethanol, cleared in xylene and mounted using Cytoseal 60 or Mounting Medium (Electron Microscopy Sciences). Specimens were documented photographically using a Nikon Eclipse E600 or 80i upright microscope and analyzed with the NIS-Elements Basic Research software.

### Prussian Blue staining of splenic MDMs

Splenocytes were grown for 5 days in chamber slide under multiple different conditions and drug treatments as indicated. These cells were fixed with Formalin (Leica) for 30 min at room temperature. Prussian blue iron stain kit (Polysciences, Inc) was used to stain the cells. Chambers were immersed in Prussian Blue Staining Solution (equal parts of 20% hydrochloric acid and 10% potassium ferrocyanide solution) for 0.5–1 h at 37 °C, washed with distilled water and counterstained with Nuclear fast red for 5 min at room temperature. Chambers were washed with PBS twice mounted in Mounting Medium (Electron Microscopy Sciences) and imaged using 80i upright microscope (Nikon) at desired magnification.

### Protein isolation and western analysis

Samples of MDMs or total liver proteins were prepared in Lysis Buffer (50 mm Tris-HCl, pH 7.5; 150 mm NaCl, 2 mm EDTA, 10% glycerol, 1% IGEPAL CA-630 and 0.5% Triton X-100) supplemented with protease inhibitors at 4 °C. Whole-cell lysates (100 μg per reaction) were mixed with an equal volume of 2× SDS–PAGE sample buffer supplemented with 10% β-Mercaptoethanol and heated for 5 min at 100 °C. Protein samples were size fractionated on 4–20% Tris-Glycine gradient gels (Lonza, Walkersville, MD, USA) using constant voltage at room temperature, transferred overnight onto Immuno-Blot PVDF membranes (Bio-Rad cat #162-0177) at 4 °C and subjected to protein blotting using the specified antibodies at vendor recommended dilutions. Secondary antibodies conjugated to horseradish peroxidase were used at a dilution of 1:10,000 (Jackson Immunochemicals, West Grove, PA, USA). Uncropped images provided in the Source Data file.

### Antibodies for western blots and immune histochemistry

Anti-LXRa rabbit polyclonal(USBiological Cat # 364027), Anti-LXRb rabbit polyclonal (Abcam Cat # ab28479), Anti-FTH1 rabbit polyclonal (Cell Signaling Cat # 3998 s), Anti-SLC40A1 rabbit polyclonal (Novus Biological Cat # NBP1-21502SS), Anti-TFR1 rabbit polyclonal (Abcam Cat # ab84036), Anti-ARG2 rabbit polyclonal (ThermoFischer Cat # PA5-27987), Anti-SLC7A2 rabbit polyclonal (ThermoFischer Cat # PA5-77552), Primary- Anti F4/80 Rat Monoclonal (Abcam Cat # ab6640), Secondary- Biotinylated rabbit anti-Rat IgG (Vector Laboratories Cat # BA-4001), Mouse IgG HRP linked whole antibody (GE Healthcare Cat # NA931V), Rabbit IgG HRP linked whole antibody (GE healthcare Cat # NA934V), Anti-F4/80 antibody [CI:A3-1] (AbcamCat # ab6640), Anti-TRF1 antibody (GeneTex Cat # GTX102596), Peroxidase AffiniPure F(ab’)_2_ Fragment Donkey Anti- Rabbit IgG (H + L) (Jackson ImmunoResearch Cat # 711-036-152), Anti-SLC40A1 antibody (Novus Biological Cat # NBP1-21502), Biotinylated Goat Anti-Rabbit IgG Antibody (Vector Laboratories Cat # BA-1000).

### Antibodies for flow cytometry

Anti CD4 Rat Monoclonal PE/Dazzle 594 (BioLegend Cat # 100566), Anti CD8a Rat Monoclonal APC-H7 (BD Biosciences Cat # 560182), Anti CD25 Rat Monoclonal APC (BioLegend Cat #101909), Anti CD69 Armenian Hamster Monoclonal BV711(BioLegend Cat # 104537), Anti Foxp3 Rat Monoclonal PE (BD BiosciencesCat # 560414), Anti CD11b Rat Monoclonal APC Cy7 (BD Biosciences Cat # 557657), Anti F4/80 Rat Monoclonal APC (BioLegend Cat # 123116), Anti Gr-1 Rat Monoclonal BV711 (BioLegend Cat # 108443), Anti CD38 Rat Monoclonal PE/Cy7 (BioLegend Cat # 102717), Anti EGR2 Rat Monoclonal (PE Life Tech Cat # 12-6691-80).

### Proteomics

Protein samples were concentrated (up to eightfold) by lyophilization and 13 μg from each sample was separated by SDS–PAGE for a distance of 1.5 cm. The entire lanes were excised and sliced into uniform 1-mm slices. Three adjacent slices were combined to generate a total of five fractions per sample. These fractions were digested with trypsin and each fraction was analyzed by LC-MS/MS on a Q Exactive HF mass spectrometer using a 120 min LC gradient. MS/MS spectra generated from a total of 30 LC-MS/MS runs were searched with full tryptic specificity against the UniProt mouse database (www.uniprot.org; 10/01/2018) using the MaxQuant 1.6.3.3 program. “Match between runs” feature was used to help transfer identifications across experiments to minimize missing values. Proteins and peptides identified only by matching (no MS/MS information) are indicated in the “Identification Type” columns. Protein quantification was performed using unique peptides. False-discovery rates for protein, and peptide identifications were set at 1%. A total of 2208 protein groups were identified, including proteins identified by a single razor + unique (nonredundant) peptide that is generally considered as low confidence identification. The abundance of a protein in a sample was determined from the sum of the peptide MS intensities for the protein. Because larger proteins will generate more peptides, the intensity values were also adjusted by normalizing against the number of theoretical peptides for each protein (iBAQ intensity) and further normalized to take into account the potential differences in sample loading using the MaxLFQ algorithm^67^. The LFQ intensity levels were log2 transformed and undetected intensities were floored to a minimum detected intensity across all proteins or a minimum across four samples in case of both replicates were undetected.

### Bioinformatics analysis

Unpaired *t* test was performed to estimate significance of difference between conditions and false-discovery rate was estimated using the procedure from ref. ^68^. Proteins that passed *p* < 0.05 threshold were considered significant (all passed FDR <25% threshold). List of known *TP53* targets were derived from ref. ^69^. Enrichment analysis of significantly differentially expressed proteins was done using QIAGEN’s Ingenuity Pathway Analysis software (IPA, QIAGEN Redwood City, www.qiagen.com/ingenuity) using “Diseases & Functions” and “Canonical Pathways” options. Functions with at least 20 differentially expressed proteins enriched at *p* < 0.001 threshold were considered. Pathways enriched at *p* < 0.05 that had at least five affected proteins were reported.

### Arginase assay

For assessment of arginase activity, an Arginase Activity Assay (MAK112-1KT, Sigma-Aldrich) was performed. 100 k mouse MDMs per sample were treated under different conditions, with or without Lm infection. After 24 h treatment cells were washed using PBS + 2 mm CaCl_2_ at 37 °C. Cells were lysed from the flasks using lysis buffer containing 10 mm Tris-HCl pH 7.4, protease inhibitor cocktail and 0.4% triton X-100. Arginase activity of the cell lysates was determined using the kit components and according to manufacturer’s instructions.

### NOS activity assay

For detection of NO production, 50-k mouse MDMs were treated under different conditions, and were incubated with a NO fluorescent probe following the manufacturer’s protocol (Cell BioLabs. Cat # STA-800-5) in 200 μl RPMI medium and FBS at 37 °C and 5% CO2 for 2 h. Cells were washed with PBS + 2 mm CaCl_2_ at 37°C. The NO fluorescent probe is oxidized to a highly fluorescent triazolo-fluorescein analog in the presence of intracellular NO, which emits green fluorescence, detected by Biotek Synergy 2 plate reader using standard fluorescein filters (excitation :485 ± 20 nm, emission: 528 ± 20 nm).

### Flow cytometry

Cells were washed with 2 ml of 1 × PBS at 1500 rpm for 5 min and then stained with 1 µl of Aqua live dead (Life Tech, Cat # L34966) for 20 min at room temperature. The cells were stained for cell surface markers with a combination of (where indicated) CD4-PE/Dazzle 594 (clone RM4-5, Biolegend, Cat # 100566), CD8a-APC-H7 (clone 53-6.7, BD Biosciences, Cat # 560182), CD25-APC (clone 3C7, Biolegend, Cat # 101909), CD69-BV711 (clone H1.2F3, Biolegend, Cat # 104537), CD11b-APC-H7 (clone M1/70, BD Biosciences, Cat # 557657), F4/80-APC (clone BM8, Biolegend, Cat # 123116), Gr-1-BV711 (clone RB6-8C5, Biolegend, Cat # 108443), CD38- PE/Cy7 (clone 90, Biolegend, Cat # 102717) for 20 min in fluorescence-activated cell sorting (FACS) buffer (1% FBS in PBS) at room temperature. Next the cells were washed with PBS, fixed and permeabilized Fixation/Permeabilization Kit (BD Biosciences Cat # 554714) for 15 min at 4 ° C. After washing them with 1 ml of 1× permeabilization buffer, intracellular proteins were stained using Foxp3- PE (clone MF23, BD Biosciences, Cat # 560414) or EGR2-PE (clone erongr2, Life Tech, Cat # 12-6691-80). Cells were washed with 1× permeabilization buffer two times. The cells were resuspended in 300 µl of 1% paraformaldehyde fixation buffer (Biolegend, Cat # B244799) in PBS. Samples were run on BD LSR II (BD Biosciences) and the data analyzed using FlowJo software. Cells were first gated for lymphocytes, neutrophils or monocytes (FSC/SSC) then singlets (FSC-A vs. FSC-H). The singlets were further analyzed for their uptake of the Live/Dead Aqua or zombie yellow stain to determine live versus dead cells. The cells were then gated for their identifying surface markers: F4/80 (macrophages), Gr-1 (neutrophils), CD3, CD4, CD8 (T lymphocytes), CD25, FoxP3 (Tregs). Gates were established in comparison to isotype controls. Gating strategy for every FACS plot shown in the Source Data File.

### Cytokine profile assay

Supernatants from MDM culture or peritoneal washes from mice were collected and centrifuged at 5000 *g* to remove cell and debris. The Proteome Profiler Mouse Cytokine Array Kit was used to compare the relative cytokine levels as per the manufacturer’s instructions. The developed dot blots were visualized on ImageQuant LAS 4000 imager (GE Healthcare Life Sciences). The relative cytokine levels were quantified from the images using ImageJ software.

### Reporting summary

Further information on research design is available in the Nature Research Reporting Summary linked to this article.

## Supplementary information

Supplementary Information

Reporting Summary

## Data Availability

The data that support the findings of this study are available from the authors on reasonable request, see author contributions for specific data sets. The mass spectrometry proteomics data have been deposited into the MassIVE (http://massive.ucsd.edu) and ProteomeXchange (http://www.proteomexchange.org) data repository with the accession number MSV000084686 and PXD016736, respectively. The source data underlying Figs. 1b–h, 2a–c, 3a–d, 4a–f, 5a–e, 6a–e, 6g–j and Supplementary Figs. 1f–h, 2a–c, d, e, 3a–c, 4a–f, 5a, b, 6a–d are provided as Source Data file. Original western blots included in the Source data file.

## Source data

Source Data
