# Author Correction: African-centric TP53 variant increases iron accumulation and bacterial pathogenesis but improves response to malaria toxin

**DOI:** 10.1038/s41467-020-15366-x

**Published:** 2020-03-19

**Authors:** Kumar Sachin Singh, Julia I-Ju Leu, Thibaut Barnoud, Prashanthi Vonteddu, Keerthana Gnanapradeepan, Cindy Lin, Qin Liu, James C. Barton, Andrew V. Kossenkov, Donna L. George, Maureen E. Murphy, Farokh Dotiwala

**Affiliations:** 1 Vaccine and Immunotherapy Center, The Wistar Institute, Philadelphia, PA 19104 USA; 2 Department of Genetics, The Raymond and Ruth Perelman School of Medicine at the University of Pennsylvania, Philadelphia, PA 19104 USA; 3 Program in Molecular and Cellular Oncogenesis, The Wistar Institute, Philadelphia, PA 19104 USA; 4 Graduate Group in Biochemistry and Molecular Biophysics, The Raymond and Ruth Perelman School of Medicine at the University of Pennsylvania, Philadelphia, PA 19104 USA; 5 Program in Immunology, Microenvironment and Metastasis, The Wistar Institute, Philadelphia, PA 19104 USA; 6 Southern Iron Disorders Center, Birmingham AL 35209 USA and Department of Medicine, University of Alabama at Birmingham, Birmingham, AL 35294 USA; 7 Bioinformatics Facility, The Wistar Institute, Philadelphia, PA 19104 USA

**Keywords:** Monocytes and macrophages, Bacterial host response, Parasite host response

Correction to: *Nature Communications* 10.1038/s41467-019-14151-9, published online 24 January 2020.

The original version of this Article contained an error in the author affiliations.

Cindy Lin was incorrectly associated with ‘Program in Molecular and Cellular Oncogenesis, The Wistar Institute, Philadelphia, PA 19104 USA’.

This has now been corrected in both the PDF and HTML versions of the Article.

