# Supplementary material for: African-centric TP53 variant increases iron accumulation and bacterial pathogenesis but improves response to malaria toxin
Source: Nat Commun. 2020 Jan 24;11:473. doi: 10.1038/s41467-019-14151-9 (PMC6981190; doi:10.1038/s41467-019-14151-9)
Supplement: Supplementary file 1 — Supplementary Information [file 41467_2019_14151_MOESM1_ESM.pdf]

Supplementary Information

African-centric TP53 variant increases iron accumulation and bacterial pathogenesis but improves response to malaria toxin.

Singh et al.

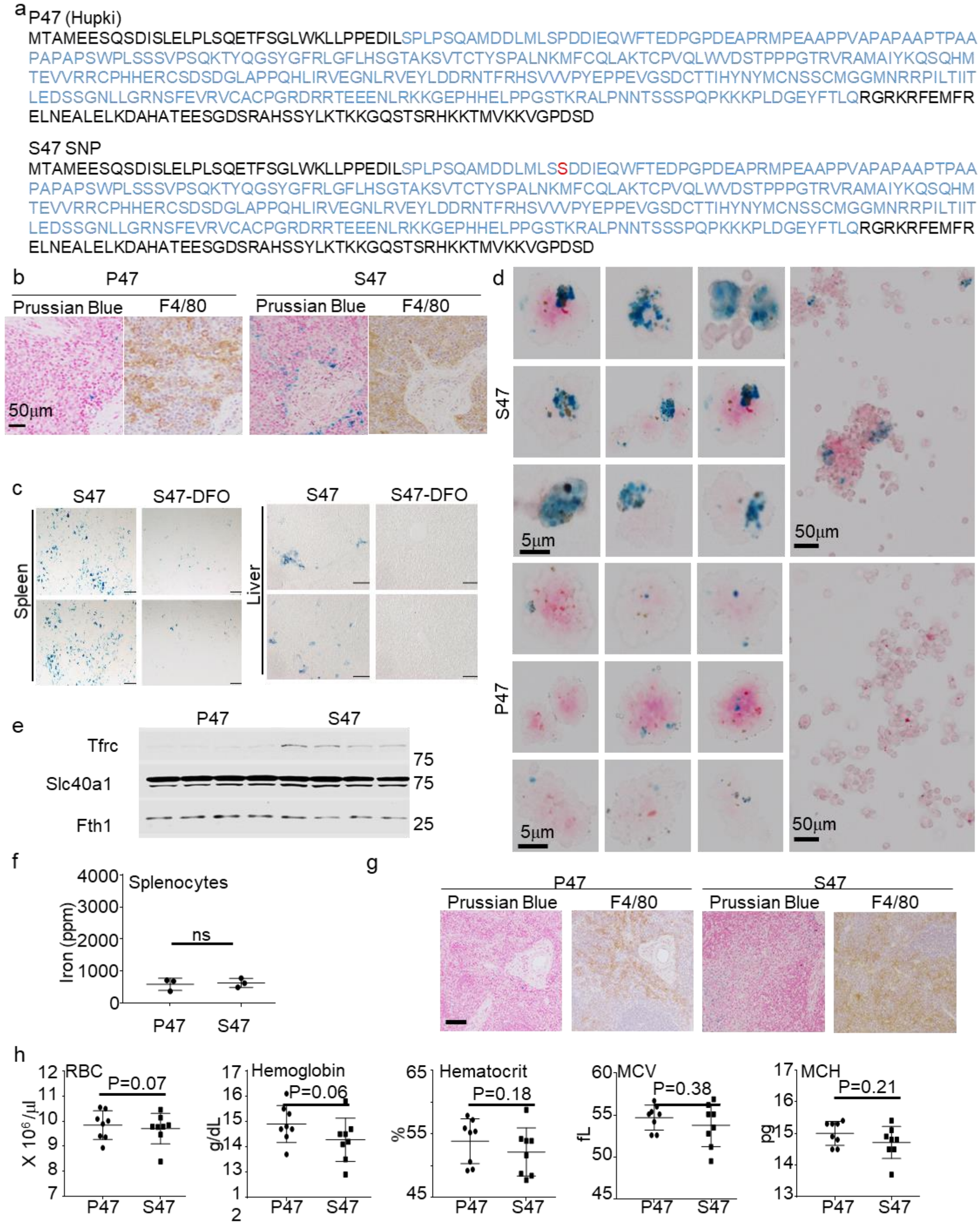

Supplementary Fig 1. S47 Hupki mice show increased iron levels in their splenic macrophages. a, *TP53* protein sequence in P47 and S47 Hupki mice. Black = mouse *TP53* exons, blue = human *TP53* exons 4-9 and Red = S47P SNP. **b,** Formalin fixed consecutive sections of male P47 and S47 mouse spleens, stained by Prussian blue and F4/80 (macrophage marker). Bar, 50 μm. (n=3 biological replicates, 5-7 fields per sample) **c,** Representative Prussian Blue stained spleen and liver sections from old S47 male mice, treated either with vehicle or 50 mg/kg DFO for 3 weeks, as indicated (n = 7 biological replicates per genotype). Bars, 50 μm. **d,** Iron accumulation in P and S47 monocyte derived macrophages by Prussian blue staining at high (left panel) and low (right panel) magnifications. (n=3 biological replicates, 20 fields per sample) Bars, 5 or 50 μm as indicated. **e,** Liver protein lysates from 12-month-old P47 and S47 male mice were analyzed by Western blot analysis for the protein indicated (n = 4 biological replicates per genotype). **f,** ICP-MS shows comparable iron content in P47 and S47 female mouse splenocytes (n=3 biological replicates). Error bars represent means ± s.e.m. ***P < 0.001, **P < 0.01, *P < 0.05, ns – not significant; by unpaired Student's t-test, relative to P47 mice. **g,** Formalin fixed sections of female P and S47 mouse spleens, stained by Prussian blue and F4/80 (macrophage marker). Bar, 50 μm. (n=3 biological replicates, 5-7 fields per sample) **h,** CBC profiles in p47 and S47 mice show trends for lower mean RBCs and hemoglobin, as well as modestly decreased hematocrit, MCV and MCH in S47 male mice than P47 mice (n=8 biological replicates). Error bars represent means ± s.e.m. P-values as indicated, by unpaired Student's t-test, relative to P47 mice. Source data are provided as a Source Data file.

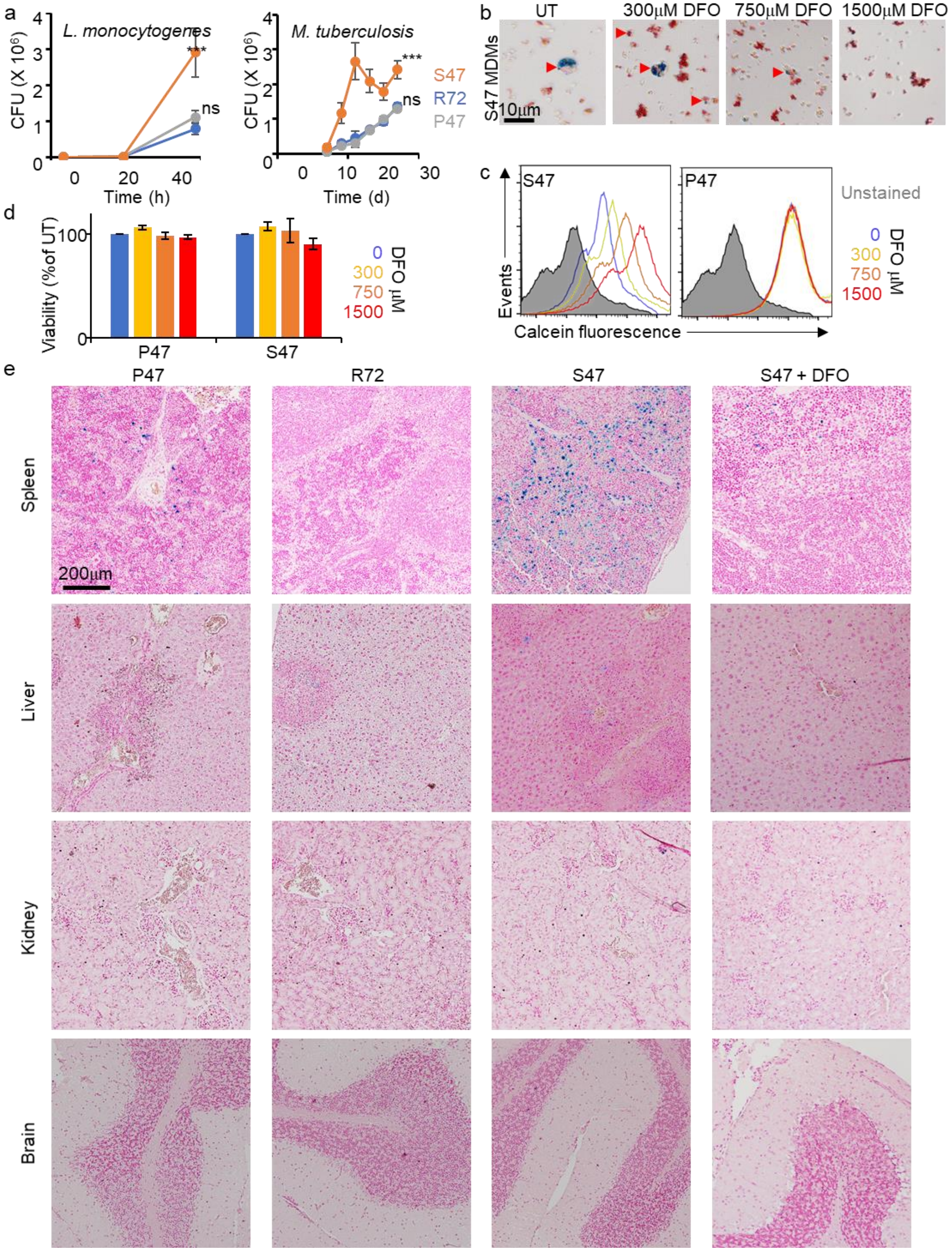

Supplementary Fig. 2. Iron content by Prussian Blue staining in tissues from S47 and P47 mice. a, MEFs of P47, R72 and S47 mice were infected with Lm (Listeria) or MTB (M. tuberculosis), and bacterial viability measured at the respective time points by CFU assay (n=6 biological replicates each with 3 technical replicates). Error bars represent means ± s.e.m. ***P<0.001, *P < 0.05, ns – not significant; by unpaired Student's t-test, relative to P47 mice. Monocyte derived macrophages (MDM) from Fig. 2c pretreated with PBS or indicated DFO concentrations show **b,** decrease in iron content by Prussian blue staining (n = 3 biological replicates, 20 fields per sample) or **c,** calcein fluorescence flow-cytometry (n = 3 biological replicates) and **d,** no significant change in MDM viability (n = 3 biological replicates with 3 technical replicates). **e,** Comparison of iron content in various organs of P47, R72, S47 and DFO treated S47 mice from Fig. 3c. DFO treatment markedly reduces iron content in S47 spleens. (n=7-10 samples per mouse, 4 fields per sample). Source data are provided as a Source Data file.

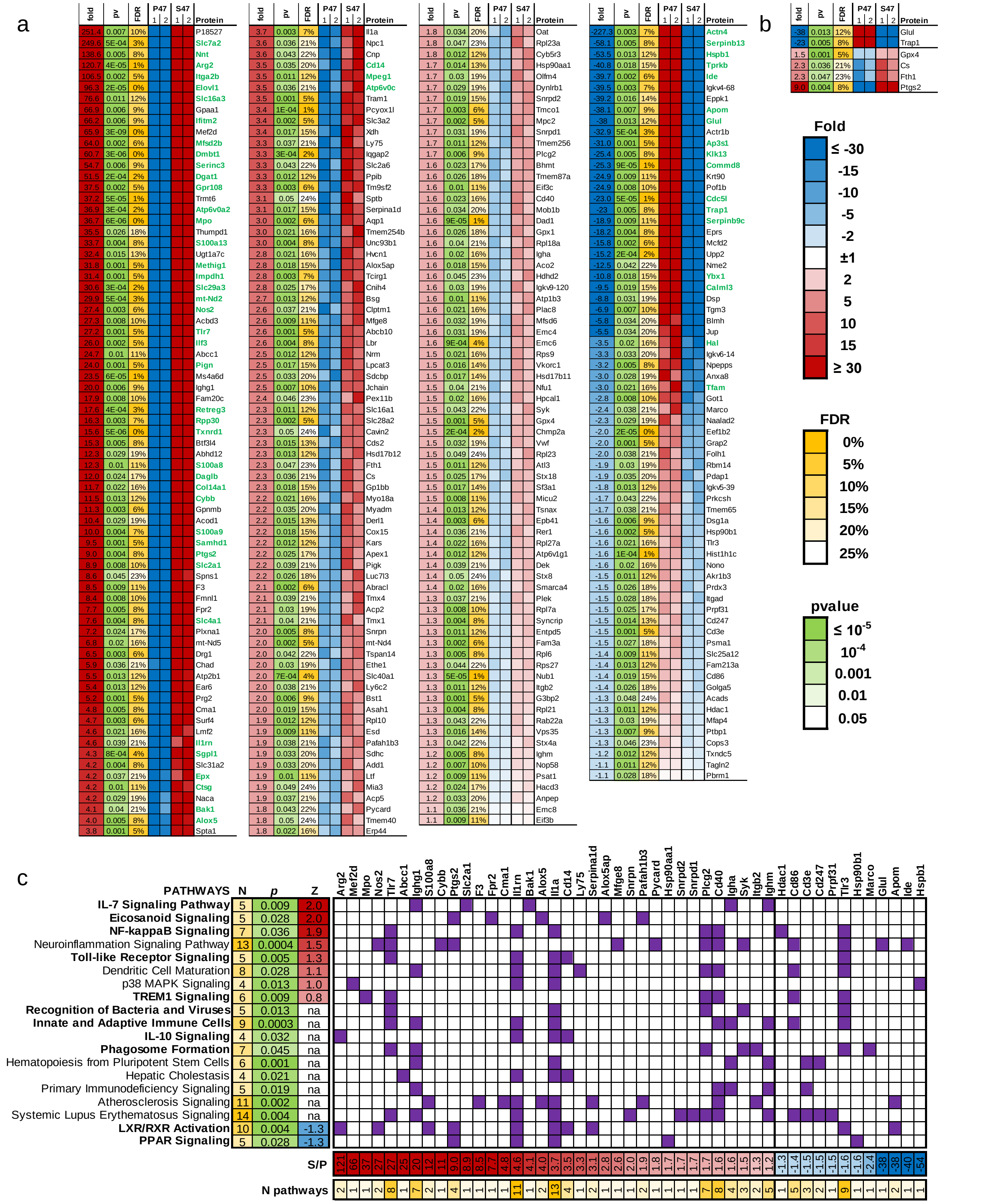

Supplementary Fig. 3. Proteomics analysis shows enrichment of innate immune pathways. a, Heatmap of the full list of significantly affected S47 proteins. P<0.05, FDR < 25%. Proteins of interest are highlighted in green. **b,** Genes involved in ferroptosis differentially expressed in S47 MDMs. P<0.05, FDR < 25%. **c,** List of significantly enriched pathways and their member proteins. Pathways of interest highlighted in bold. Z-scores predicted by Ingenuity Pathway Analysis indicate the effect on the function and pathway. Z > 0 – activated in S47 MDMs, Z < 0 – inhibited, na=not called. Source data are provided as a Source Data file.

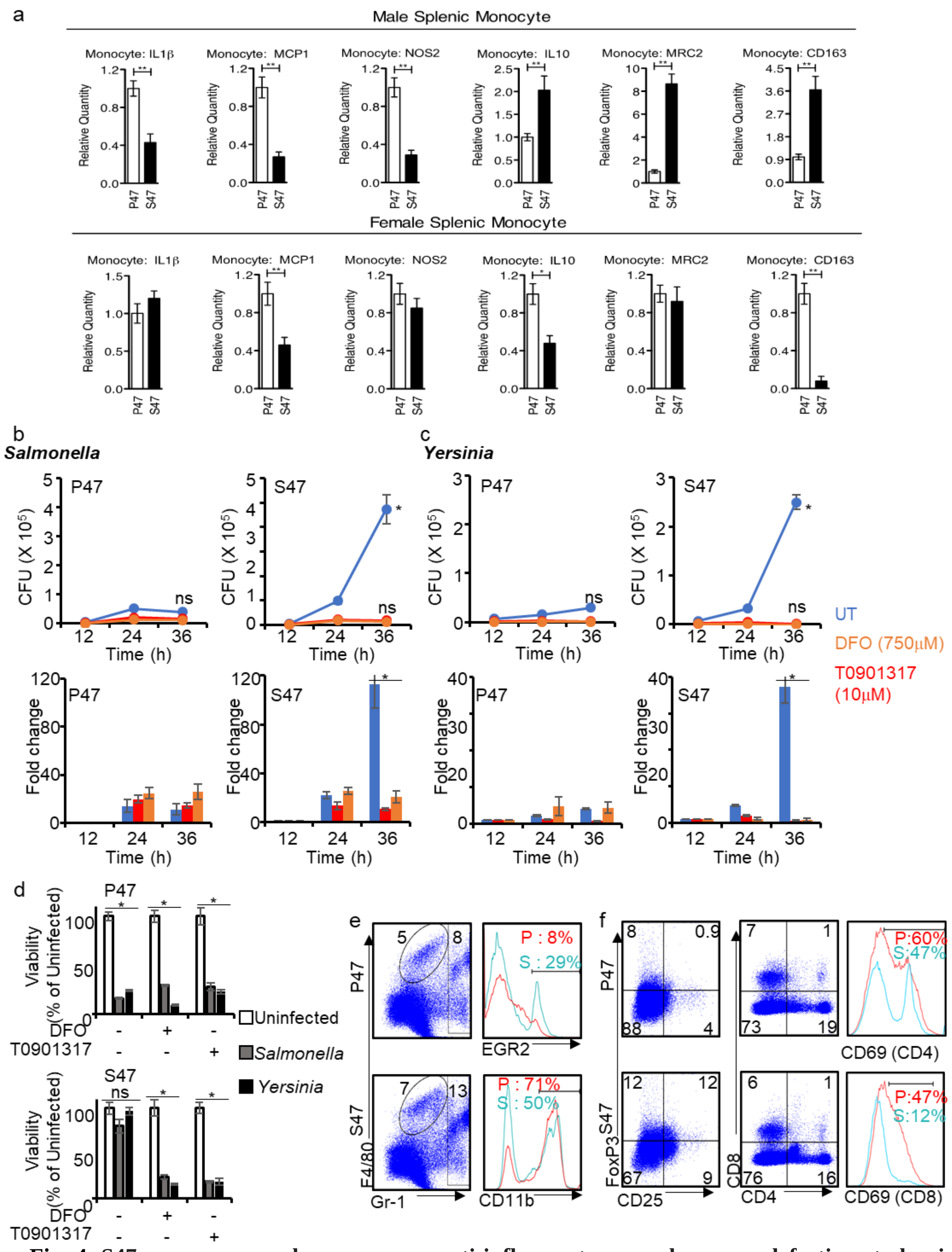

Supplementary Fig. 4. S47 mouse macrophages express anti-inflammatory markers, are defective at clearing *Salmonella* and *Yersinia* infections and exhibit anti-inflammatory response to hemozoin. a, mRNA levels of pro and anti-inflammatory markers in healthy P47 (white) and S47 (black) macrophages from male (top panel) or female (bottom panel) mice, measured by qRT-PCR (n=3). Error bars represent means ± s.e.m. **P < 0.01, *P < 0.05, by unpaired Student's t-test, relative to P47 mice. Monocyte derived macrophages (n=3 biological replicates with 3-6 technical replicates) from spleens of P47 and S47 mice were infected with **b,** *Salmonella* or **c,** *Yersinia*, and bacterial viability measured at the respective time points by CFU assay. Top panel – raw values, bottom panel fold change over 0h post infection. Error bars represent means ± s.e.m. ***P<0.001, *P < 0.05, ns – not significant; by unpaired Student's t-test, relative to P47 mice. **d,** Macrophage viability in uninfected (white bars) or Salmonella (grey bars) or Yersinia (black bars) at the last time point after infection, was measured by Resazurine cell viability assay (n=3 biological replicates with 3-6 technical replicates). Error bars represent means ± s.e.m. ***P<0.001, P < 0.01, *P < 0.05, ns – not significant; by unpaired Student's t-test, relative to the uninfected counterpart. Splenocytes from S47 and P47 mice treated with hemozoin for 8h and analyzed for the presence of **e,** macrophages (F4/80+) and neutrophils (Gr-1+). F4/80+ macrophages checked for expression of Egr2 (M2 marker) and Gr-1+ neutrophils checked for CD11b (activation marker) **f,** Tregs (CD25+, FoxP3+), CD4 and CD8 T cells. Activation of CD8 and CD4 T cells checked by CD69 levels. Source data provided in Source Data file.

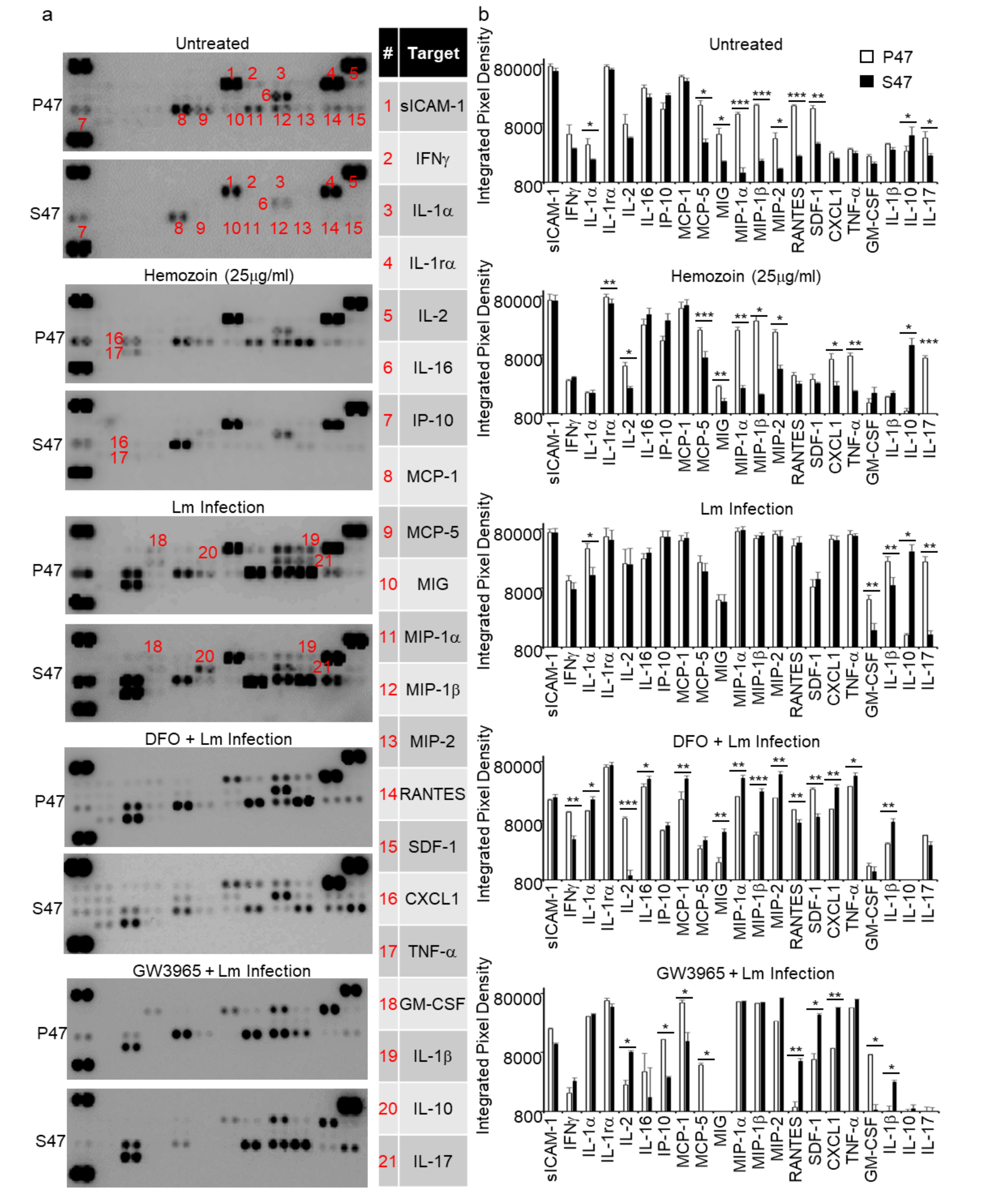

Supplementary Fig. 5. S47 mouse MDMs anti-inflammatory response to malarial pigment hemozoin and Lm infection is dependent on iron. a, Comparison of 40 mouse cytokines released in the culture medium by P47 and S47 mouse MDMs, 12 h post indicated treatments. The table maps the respective cytokines on the dot-blot array. **b,** Relative cytokine levels quantified from the dot blots (n=3). Error bars represent means ± s.e.m. ***P<0.001, **P<0.01, *P < 0.05, unlabeled - not significant; by unpaired Student's t-test, relative to P47 mice. Source data are provided as a Source Data file.

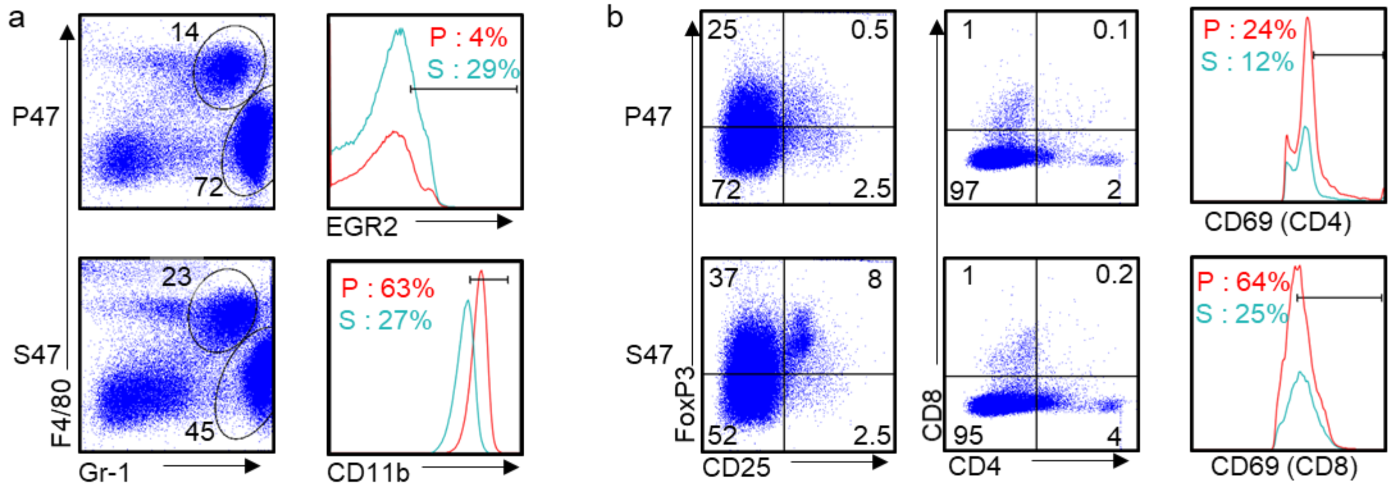

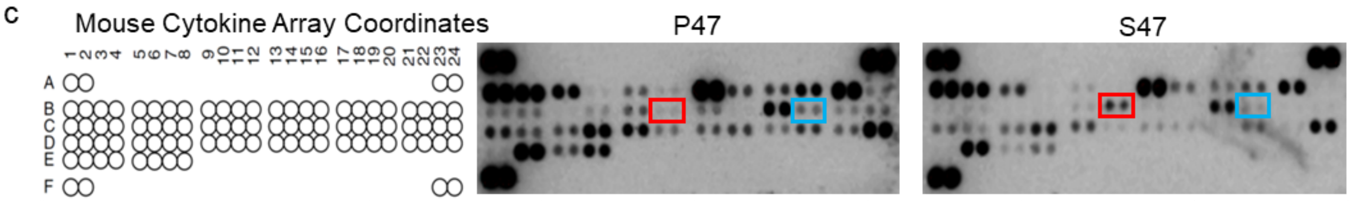

Coordinate	Target	Coordinate	Target	Coordinate	Target	Coordinate	Target
B1, B2	BLC	C1, C2	IL-3	D1, D2	IP-10	E1, E3	TARC
B3, B4	C5/C5a	C3, C4	IL-4	D3, D4	CXCL11	E3, E4	TIMP-1
B5, B6	G-CSF	C5, C6	IL-5	D5, D6	CXCL1	E5, E6	TNF-α
B7, B8	GM-CSF	C7, C8	IL-6	D7, D8	M-CSF	E7, E8	TREM-1
B9, B10	I-309	C9, C10	IL-7	D9, D10	MCP-1		
B11, B12	Eotaxin	C11, C12	IL-10	D11, D12	MCP-5		
B13, B14	sICAM-1	C13, C14	IL-13	D13, D14	MIG		
B15, B16	IFN-γ	C15, C16	IL-12	D15, D16	MIP-1α		
B17, B18	IL-1α	C17, C18	IL-16	D17, D18	MIP-1β	A1, A2	Ref. Spot
B19, B20	IL-1β	C19, C20	IL-17	D19, D20	MIP-2	A3, A4	
B21, B22	IL-1ra	C21, C22	IL-23	D21, D22	CCL5	F1, F2	
B23, B24	IL-2	C23, C24	IL-27	D23, D24	SDF-1	F23, F24	(-) Control

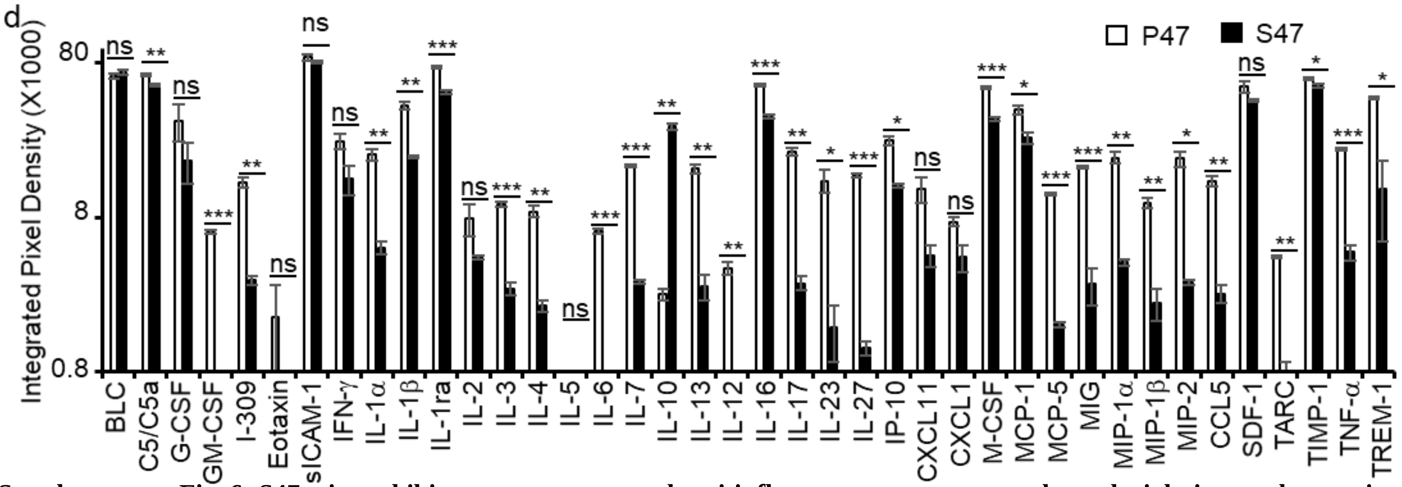

Supplementary Fig. 6: S47 mice exhibit a more pronounced anti-inflammatory response to the malarial pigment hemozoin. Cells from peritoneal washes of S47 and P47 mice 12 h post IP Hemozoin injection, analyzed for **a,** the presence of macrophages (F4/80+), neutrophils (Gr-1+), **b,** Tregs (CD25+, FoxP3+), CD4 and CD8 T cells. F4/80+ macrophages checked for expression of Egr2 (M2 marker) and Gr-1+ neutrophils checked for CD11b (activation marker). Activation of CD8 and CD4 T cells checked by CD69 levels. **c,** Release of 40 mouse cytokines in the peritoneum of P47 and S47 mice, compared 12 h post Hemozoin injection. The table maps the respective cytokines on the dot-blot array. Red boxes: IL10, Blue boxes: IL17. **d,** Relative cytokine levels quantified from the dot blots (n=5). Error bars represent means ± s.e.m. ***P<0.001, **P<0.01, *P < 0.05, ns – not significant; by unpaired Student’s t-test, relative to P47 mice. Source data are provided as a Source Data file.
