# Supplementary material for: African-centric TP53 variant increases iron accumulation and bacterial pathogenesis but improves response to malaria toxin
Source: Nat Commun. 2020 Jan 24;11:473. doi: 10.1038/s41467-019-14151-9 (PMC6981190; doi:10.1038/s41467-019-14151-9)
Supplement: Supplementary file 2 — Reporting Summary [file 41467_2019_14151_MOESM2_ESM.pdf]

nature research

Corresponding author(s): Farokh Dotiwala
Maureen E. Murphy
Donna L. George

Last updated by author(s): Dec 13, 2019

Reporting Summary

Nature Research wishes to improve the reproducibility of the work that we publish. This form provides structure for consistency and transparency in reporting. For further information on Nature Research policies, seeAuthors & Referees and theEditorial Policy Checklist .

Statistics

For all statistical analyses, confirm that the following items are present in the figure legend, table legend, main text, or Methods section.

n/a	Confirmed	
☐	☒	The exact sample size (*n*) for each experimental group/condition, given as a discrete number and unit of measurement
☐	☒	A statement on whether measurements were taken from distinct samples or whether the same sample was measured repeatedly
☐	☒	The statistical test(s) used AND whether they are one- or two-sided<br>*Only common tests should be described solely by name; describe more complex techniques in the Methods section.*
☒	☐	A description of all covariates tested
☒	☐	A description of any assumptions or corrections, such as tests of normality and adjustment for multiple comparisons
☐	☒	A full description of the statistical parameters including central tendency (e.g. means) or other basic estimates (e.g. regression coefficient) AND variation (e.g. standard deviation) or associated estimates of uncertainty (e.g. confidence intervals)
☒	☐	For null hypothesis testing, the test statistic (e.g. *F*, *t*, *r*) with confidence intervals, effect sizes, degrees of freedom and *P* value noted<br>*Give P values as exact values whenever suitable.*
☒	☐	For Bayesian analysis, information on the choice of priors and Markov chain Monte Carlo settings
☒	☐	For hierarchical and complex designs, identification of the appropriate level for tests and full reporting of outcomes
☒	☐	Estimates of effect sizes (e.g. Cohen's *d*, Pearson's *r*), indicating how they were calculated

Our web collection on statistics for biologists contains articles on many of the points above.

Software and code

Policy information about availability of computer code

Data collection	NIS-Elements Basic Research Nikon<br>FlowJo FlowJo LLC
Data analysis	MS-Excel, Office, PowerPoint Microsoft Inc<br>Prism 7 Graph Pad Inc<br>MaxQuant 16.3.3 Max Planck Institute<br>MaxLFQ Max Planck Institute<br>QuantStudio 7 Flex Real-Time PCR System ThermoFisher<br>Image J 1.52a National Institutes of Health<br>Matlab R2016a MathWorks<br>Ingenuity Pathway Analysis Qiagen<br>Adobe Illustrator CS6 Adobe

For manuscripts utilizing custom algorithms or software that are central to the research but not yet described in published literature, software must be made available to editors/reviewers. We strongly encourage code deposition in a community repository (e.g. GitHub). See the Nature Research guidelines for submitting code & software for further information.

Data

Policy information about availability of data

All manuscripts must include a data availability statement. This statement should provide the following information, where applicable:

- Accession codes, unique identifiers, or web links for publicly available datasets
- A list of figures that have associated raw data
- A description of any restrictions on data availability

All our figures have associated raw data available and will be provided at the time of final submission.

Field-specific reporting

Please select the one below that is the best fit for your research. If you are not sure, read the appropriate sections before making your selection.

[x] Life sciences [ ] Behavioural & social sciences [ ] Ecological, evolutionary & environmental sciences

For a reference copy of the document with all sections, see nature.com/documents/nr-reporting-summary-flat.pdf

Life sciences study design

All studies must disclose on these points even when the disclosure is negative.

Sample size	Sample size calculated based on statistics of previous bacteria infection studies. WT and S47, male mice at 8 weeks age were randomly assigned to the experimental groups. A group size of n = 10 mice (unless otherwise mentioned) provided 90% power to detect 2 to 10-fold increase in Hp infection in S47 mice. DNA of African Americans with elevated saturated serum transferrin levels, for human studies was obtained randomized from the HEIRS study.
Data exclusions	No data excluded
Replication	All experiments were done in triplicate or more where mentioned in order to verify the reproducibility of the data.
Randomization	DNA samples from 479 African Americans with elevated transferrin saturation and serum ferritin were compared with (OR 1.68 (95% CI 1.07-2.65) p=0.023) Male mice were used for all studies once data revealed only modest differences in iron levels and anti-inflammatory polarization between WT and S47 female mice. Controls and experimental groups were age and genotype-matched non-littermates.
Blinding	Blinding was achieved by separation of research staff that collected and processed the specimens from the staff that analyzed the de-identified specimens by Flow, microscopy, etc.

Reporting for specific materials, systems and methods

We require information from authors about some types of materials, experimental systems and methods used in many studies. Here, indicate whether each material, system or method listed is relevant to your study. If you are not sure if a list item applies to your research, read the appropriate section before selecting a response.

Materials & experimental systems

n/a	Involved in the study	
[ ]	[x]	Antibodies
[ ]	[x]	Eukaryotic cell lines
[ ]	[ ]	Palaeontology
[ ]	[x]	Animals and other organisms
[ ]	[x]	Human research participants
[ ]	[ ]	Clinical data

Methods

n/a	Involved in the study	
[ ]	[ ]	ChIP-seq
[ ]	[x]	Flow cytometry
[ ]	[ ]	MRI-based neuroimaging

Antibodies

Antibodies used	Antibodies for WB and IHC:<br>Anti-LXRa rabbit polyclonal USBiological Cat # 364027<br>Anti-LXRb rabbit polyclonal Abcam Cat # ab28479<br>Anti-FTH1 rabbit polyclonal Cell Signalling Cat # 3998S<br>Anti-SLC40A1 rabbit polyclonal Novus Biological Cat # NBP1-21502SS<br>Anti-TFR1 rabbit polyclonal Abcam Cat # ab84036

Anti-ARG2 rabbit polyclonal ThermoFischer Cat # PA5-27987
Anti-SLC7A2 rabbit polyclonal ThermoFischer Cat # PA5-77552
Primary- Anti F4/80 Rat Monoclonal Abcam Cat# ab6640
Secondary- Biotinylated rabbit anti-Rat IgG Vector Laboratories Cat# BA-4001
Mouse IgG HRP linked whole antibody GE Healthcare Cat # NA931V
Rabbit IgG HRP linked whole antibody GE healthcare Cat # NA934V
Anti-F4/80 antibody [CI:A3-1] Abcam Cat# ab6640
Anti-TRF1 antibody GeneTex Cat # GTX102596
Peroxidase AffiniPure F(ab')$_2$ Fragment Donkey Anti- Rabbit IgG (H+L) Jackson ImmunoResearch Cat # 711-036-152
Anti-SLC40A1 antibody Novus Biological Cat # NBP1-21502
Biotinylated Goat Anti-Rabbit IgG Antibody Vector Laboratories Cat # BA-1000

Antibodies for FACS:
Anti CD4 Rat Monoclonal PE/Dazzle 594 BioLegend Cat # 100566
Anti CD8a Rat Monoclonal APC-H7 BD Biosciences Cat # 560182
Anti CD25 Rat Monoclonal APC BioLegend Cat # 101909
Anti CD69 Armenian Hamster Monoclonal BV711 BioLegend Cat # 104537
Anti Foxp3 Rat Monoclonal PE BD Biosciences Cat # 560414
Anti CD11b Rat Monoclonal APC Cy7 BD Biosciences Cat # 557657
Anti F4/80 Rat Monoclonal APC BioLegend Cat # 123116
Anti Gr-1 Rat Monoclonal BV711 BioLegend Cat # 108443
Anti CD38 Rat Monoclonal PE/Cy7 BioLegend Cat # 102717
Anti EGR2 Rat Monoclonal PE Life Tech Cat # 12-6691-80

Antibodies for FACS compensation:
Anti CD3 Mouse Monoclonal PE/Dazzle 594 BioLegend Cat # 317346
Anti CD3 Mouse Monoclonal APC BioLegend Cat # 300412
Anti CD3 Mouse Monoclonal APC Cy7 BioLegend Cat # 300317
Anti CD3 Mouse Monoclonal BV711 BioLegend Cat # 344838
Anti CD3 Mouse Monoclonal PE BioLegend Cat # 300408
Anti CD3 Mouse Monoclonal PE Cy7 BioLegend Cat # 300316

Validation

All antibodies were validated by the manufacturer.

Eukaryotic cell lines

Policy information about cell lines

Cell line source(s)

P47 Human LCL from B lymphocyte (Deidentified) Coriell Institute Cat # GM18870
S47 Human LCL from B lymphocyte (Deidentified) Coriell Institute Cat # GM18871
Mouse embryonic fibroblasts Murphy Lab
Mouse Splenocytes/MDMs Murphy/Dotiwala Labs

Authentication

LCLs were authenticated by Coriell
MEFs and Mouse MDMs were authenticated by PCR for WT and S47 TP53 alleles and by cell surface markers for monocytes.

Mycoplasma contamination

All cell lines were confirmed to be free of mycoplasma contamination

Commonly misidentified lines
(See ICLAC register)

Not used

Palaeontology

Specimen provenance

Provide provenance information for specimens and describe permits that were obtained for the work (including the name of the issuing authority, the date of issue, and any identifying information).

Specimen deposition

Indicate where the specimens have been deposited to permit free access by other researchers.

Dating methods

If new dates are provided, describe how they were obtained (e.g. collection, storage, sample pretreatment and measurement), where they were obtained (i.e. lab name), the calibration program and the protocol for quality assurance OR state that no new dates are provided.

☐ Tick this box to confirm that the raw and calibrated dates are available in the paper or in Supplementary Information.

Animals and other organisms

Policy information about studies involving animals; ARRIVE guidelines recommended for reporting animal research

Laboratory animals	Wild-type (WT) and S47 humanized TP53 knock-in (Hupki) mice were generated by Dr. Maureen Murphy (The Wistar Institute, Philadelphia, PA). All mice were backcrossed to C57bBl/6 for >10 generations, and sibling littermates were used for majority of the analyses. Mice were housed in plastic cages with ad libitum diet and maintained with a 12-hr light/12-hr dark cycle at 22°C. Male mice were used for all studies once data revealed modest differences in iron levels between WT and S47 female mice. Controls and experimental groups were age and genotype-matched non-littermates.
Wild animals	no wild animals involved
Field-collected samples	no field samples collected
Ethics oversight	All protocols were approved by The Wistar Institute and the Perelman School of Medicine at the University of Pennsylvania, Institutional Animal Care and Use Committee (IACUC)

Note that full information on the approval of the study protocol must also be provided in the manuscript.

Human research participants

Policy information about studies involving human research participants

Population characteristics	We analyzed the TP53 SNP rs1800371 in 479 African American samples, both men and women who were HEIRS Study participants ≥25 years of age. Samples were obtained from participants after they provided informed consent. All participants had elevated transferrin saturation (>50%); in approximately one quarter of the cases, participants also had serum ferritin values >300 µg/mL. DNA samples or buffy coat punches were obtained from the HEIRS Study, part of the BioLINCC repository of the National Heart Lung and Blood Institute.
Recruitment	No participants were recruited for this study. Instead de-identified DNA samples from HEIRS study were obtained.
Ethics oversight	Since the samples are de-identified, this study is classified as exemption 4 and was approved by the Wistar IRB.

Note that full information on the approval of the study protocol must also be provided in the manuscript.

Clinical data

Policy information about clinical studies

All manuscripts should comply with the ICMJEguidelines for publication of clinical research and a completedCONSORT checklist must be included with all submissions.

Clinical trial registration	*Provide the trial registration number from ClinicalTrials.gov or an equivalent agency.*
Study protocol	*Note where the full trial protocol can be accessed OR if not available, explain why.*
Data collection	*Describe the settings and locales of data collection, noting the time periods of recruitment and data collection.*
Outcomes	*Describe how you pre-defined primary and secondary outcome measures and how you assessed these measures.*

ChIP-seq

Data deposition

☐ Confirm that both raw and final processed data have been deposited in a public database such as GEO.

☐ Confirm that you have deposited or provided access to graph files (e.g. BED files) for the called peaks.

Data access links<br>*May remain private before publication.*	*For "Initial submission" or "Revised version" documents, provide reviewer access links. For your "Final submission" document, provide a link to the deposited data.*
Files in database submission	*Provide a list of all files available in the database submission.*
Genome browser session<br>(e.g. UCSC)	*Provide a link to an anonymized genome browser session for "Initial submission" and "Revised version" documents only, to enable peer review. Write "no longer applicable" for "Final submission" documents.*

Methodology

Replicates	*Describe the experimental replicates, specifying number, type and replicate agreement.*
Sequencing depth	*Describe the sequencing depth for each experiment, providing the total number of reads, uniquely mapped reads, length of reads and whether they were paired- or single-end.*

Antibodies	*Describe the antibodies used for the ChIP-seq experiments; as applicable, provide supplier name, catalog number, clone name, and lot number.*
Peak calling parameters	*Specify the command line program and parameters used for read mapping and peak calling, including the ChIP, control and index files used.*
Data quality	*Describe the methods used to ensure data quality in full detail, including how many peaks are at FDR 5% and above 5-fold enrichment.*
Software	*Describe the software used to collect and analyze the ChIP-seq data. For custom code that has been deposited into a community repository, provide accession details.*

Flow Cytometry

Plots

Confirm that:

- [x] The axis labels state the marker and fluorochrome used (e.g. CD4-FITC).
- [x] The axis scales are clearly visible. Include numbers along axes only for bottom left plot of group (a 'group' is an analysis of identical markers).
- [x] All plots are contour plots with outliers or pseudocolor plots.
- [x] A numerical value for number of cells or percentage (with statistics) is provided.

Methodology

Sample preparation	Cells were washed with 2ml of 1X PBS at 1500 rpm for 5 min and then stained with 1 ul of Aqua live dead (Life Tech, Cat # L34966) for 20 min at room temperature. The cells were stained for cell surface markers with a combination of (where indicated) CD4- PE/Dazzle 594 (clone RM4-5, Biolegend, Cat # 100566), CD8a-APC-H7 (clone 53-6.7, BD Biosciences, Cat # 560182), CD25-APC (clone 3C7, Biolegend, Cat # 101909), CD69-BV711 (clone H1.2F3, Biolegend, Cat # 104537), CD11b-APC-H7 (clone M1/70, BD Biosciences, Cat # 557657), F4/80-APC (clone BM8, Biolegend, Cat # 123116), Gr-1-BV711 (clone RB6-8C5, Biolegend, Cat # 108443), CD38- PE/Cy7 (clone 90, Biolegend, Cat # 102717) for 20 min in FACS buffer (1% FBS in PBS) at room temperature. Next the cells were washed with PBS, fixed and permeabilized Fixation/Permeabilization Kit (BD Biosciences Cat # 554714) for 15 min at 4°C. After washing them with 1 ml of 1X permeabilization buffer, intracellular proteins were stained using Foxp3- PE (clone MF23, BD Biosciences, Cat # 560414) or EGR2-PE (clone erongr2, Life Tech, Cat # 12-6691-80). Cells were washed with 1X permeabilization buffer 2 times. The cells were resuspended in 300 ul of 1% paraformaldehyde fixation buffer (Biolegend, Cat # B244799) in PBS. Samples were run on BD LSR II (BD Biosciences) and the data analyzed using FlowJo software.
Instrument	BD LSR II (BD Biosciences)
Software	FlowJo
Cell population abundance	200k-1million cells per sample were used for Flow cytometry
Gating strategy	Cells were first gated for lymphocytes, neutrophils or monocytes (FSC/SSC) then singlets (FSC-A vs. FSC-H). The singlets were further analyzed for their uptake of the Live/Dead Aqua or zombie yellow stain to determine live versus dead cells. The cells were then gated for their identifying surface markers: F4/80 (macrophages), Gr-1 (neutrophils), CD3, CD4, CD8 (T lymphocytes), CD25, FoxP3 (Tregs).

- [x] Tick this box to confirm that a figure exemplifying the gating strategy is provided in the Supplementary Information.

Magnetic resonance imaging

Experimental design

Design type	*Indicate task or resting state; event-related or block design.*
Design specifications	*Specify the number of blocks, trials or experimental units per session and/or subject, and specify the length of each trial or block (if trials are blocked) and interval between trials.*
Behavioral performance measures	*State number and/or type of variables recorded (e.g. correct button press, response time) and what statistics were used to establish that the subjects were performing the task as expected (e.g. mean, range, and/or standard deviation across subjects).*

Acquisition

Imaging type(s) — *Specify: functional, structural, diffusion, perfusion.*

Field strength — *Specify in Tesla*

Sequence & imaging parameters — *Specify the pulse sequence type (gradient echo, spin echo, etc.), imaging type (EPI, spiral, etc.), field of view, matrix size, slice thickness, orientation and TE/TR/flip angle.*

Area of acquisition — *State whether a whole brain scan was used OR define the area of acquisition, describing how the region was determined.*

Diffusion MRI ☐ Used ☐ Not used

Preprocessing

Preprocessing software — *Provide detail on software version and revision number and on specific parameters (model/functions, brain extraction, segmentation, smoothing kernel size, etc.).*

Normalization — *If data were normalized/standardized, describe the approach(es): specify linear or non-linear and define image types used for transformation OR indicate that data were not normalized and explain rationale for lack of normalization.*

Normalization template — *Describe the template used for normalization/transformation, specifying subject space or group standardized space (e.g. original Talairach, MNI305, ICBM152) OR indicate that the data were not normalized.*

Noise and artifact removal — *Describe your procedure(s) for artifact and structured noise removal, specifying motion parameters, tissue signals and physiological signals (heart rate, respiration).*

Volume censoring — *Define your software and/or method and criteria for volume censoring, and state the extent of such censoring.*

Statistical modeling & inference

Model type and settings — *Specify type (mass univariate, multivariate, RSA, predictive, etc.) and describe essential details of the model at the first and second levels (e.g. fixed, random or mixed effects; drift or auto-correlation).*

Effect(s) tested — *Define precise effect in terms of the task or stimulus conditions instead of psychological concepts and indicate whether ANOVA or factorial designs were used.*

Specify type of analysis: ☐ Whole brain ☐ ROI-based ☐ Both

Statistic type for inference
(See Eklund et al. 2016) — *Specify voxel-wise or cluster-wise and report all relevant parameters for cluster-wise methods.*

Correction — *Describe the type of correction and how it is obtained for multiple comparisons (e.g. FWE, FDR, permutation or Monte Carlo).*

Models & analysis

n/a	Involved in the study
☐	☐ Functional and/or effective connectivity
☐	☐ Graph analysis
☐	☐ Multivariate modeling or predictive analysis

Functional and/or effective connectivity — *Report the measures of dependence used and the model details (e.g. Pearson correlation, partial correlation, mutual information).*

Graph analysis — *Report the dependent variable and connectivity measure, specifying weighted graph or binarized graph, subject- or group-level, and the global and/or node summaries used (e.g. clustering coefficient, efficiency, etc.).*

Multivariate modeling and predictive analysis — *Specify independent variables, features extraction and dimension reduction, model, training and evaluation metrics.*
